# Design of an interface to communicate artificial intelligence-based prognosis for patients with advanced solid tumors: a user-centered approach

**DOI:** 10.1093/jamia/ocad201

**Published:** 2023-10-17

**Authors:** Catherine J Staes, Anna C Beck, George Chalkidis, Carolyn H Scheese, Teresa Taft, Jia-Wen Guo, Michael G Newman, Kensaku Kawamoto, Elizabeth A Sloss, Jordan P McPherson

**Affiliations:** College of Nursing, University of Utah, Salt Lake City, UT 84112, United States; Department of Biomedical Informatics, School of Medicine, University of Utah, Salt Lake City, UT 84108, United States; Department of Internal Medicine, Huntsman Cancer Institute, University of Utah, Salt Lake City, UT 84112, United States; Healthcare IT Research Department, Center for Digital Services, Hitachi Ltd., Tokyo, Japan; College of Nursing, University of Utah, Salt Lake City, UT 84112, United States; Department of Biomedical Informatics, School of Medicine, University of Utah, Salt Lake City, UT 84108, United States; Department of Biomedical Informatics, School of Medicine, University of Utah, Salt Lake City, UT 84108, United States; College of Nursing, University of Utah, Salt Lake City, UT 84112, United States; Department of Biomedical Informatics, School of Medicine, University of Utah, Salt Lake City, UT 84108, United States; Department of Population Sciences, Huntsman Cancer Institute, Salt Lake City, UT 84112, United States; Department of Biomedical Informatics, School of Medicine, University of Utah, Salt Lake City, UT 84108, United States; College of Nursing, University of Utah, Salt Lake City, UT 84112, United States; Department of Pharmacotherapy, College of Pharmacy, University of Utah, Salt Lake City, UT 84108, United States; Department of Pharmacy, Huntsman Cancer Institute, Salt Lake City, UT 84112, United States

**Keywords:** data visualization, artificial intelligence, user-centered design, neoplasms/mortality, prognosis, clinical decision-making

## Abstract

**Objectives:**

To design an interface to support communication of machine learning (ML)-based prognosis for patients with advanced solid tumors, incorporating oncologists’ needs and feedback throughout design.

**Materials and Methods:**

Using an interdisciplinary user-centered design approach, we performed 5 rounds of iterative design to refine an interface, involving expert review based on usability heuristics, input from a color-blind adult, and 13 individual semi-structured interviews with oncologists. Individual interviews included patient vignettes and a series of interfaces populated with representative patient data and predicted survival for each treatment decision point when a new line of therapy (LoT) was being considered. Ongoing feedback informed design decisions, and directed qualitative content analysis of interview transcripts was used to evaluate usability and identify enhancement requirements.

**Results:**

Design processes resulted in an interface with 7 sections, each addressing user-focused questions, supporting oncologists to “tell a story” as they discuss prognosis during a clinical encounter. The iteratively enhanced interface both triggered and reflected design decisions relevant when attempting to communicate ML-based prognosis, and exposed misassumptions. Clinicians requested enhancements that emphasized interpretability over explainability. Qualitative findings confirmed that previously identified issues were resolved and clarified necessary enhancements (eg, use months not days) and concerns about usability and trust (eg, address LoT received elsewhere). Appropriate use should be in the context of a conversation with an oncologist.

**Conclusion:**

User-centered design, ongoing clinical input, and a visualization to communicate ML-related outcomes are important elements for designing any decision support tool enabled by artificial intelligence, particularly when communicating prognosis risk.

## Background and significance

Artificial intelligence (AI) is increasingly used in health care to perform tasks that typically require human intelligence.^1–3^ Machine learning (ML), a common AI method, allows algorithms to learn from data and experience, and may be used to analyze electronic health records (EHRs) to screen patients, diagnose, and predict outcomes.^2^^,^^4^^,^^5^ These methods can improve upon expert human performance,^6^^,^^7^ often involving less time or resources.^1^ However, use of AI in health care raises practical, regulatory, and ethical concerns related to quality, disclosure, scalability, trust, governance, deployment, appropriateness, and impact on patient–provider interaction.^1,4^,^8–18^ It is especially difficult when AI is used in clinical decision support (CDS) for prognosis and treatment decisions because, unlike diagnostic decisions, the decisions may not be linked to a gold standard, such as biopsy.^19^ Integration of AI in health care requires these concerns be addressed with a design that supports, not replaces, communication and decision-making among care teams and patients.^1^^,^^4^^,^^20^

Communication about prognosis is particularly important when treating patients with advanced cancer. Prognosis, a key component of a serious illness conversation (SIC), is needed to assess whether there is “no strong evidence supporting the clinical value of further anticancer treatment,” a criteria from the Choosing Wisely initiative.^21^ When chemotherapy is likely futile, high-value care may prioritize supportive care over cancer-treating regimens that no longer prolong life nor align with goals.^22–25^ Further, early palliative care has been associated with higher quality of life and longer survival.^26^ Understanding prognosis is critical for delivering timely, guideline-based palliative and supportive care^21^,^27–31^; however, a recent study found only 55% of patients who want prognostic information receive it.^32^ Similarly, providers need support, as they often overestimate survival^33^^,^^34^ and use aggressive care at end of life (EOL).^35^^,^^36^ When approaching the final months of life, patients and caregivers must understand available options and resources to make informed care decisions for evolving goals.

Prognostication is amenable to ML-based AI, and our team and others have developed models to predict 6-month survival among persons with cancer.^37–40^ ML prediction has been used to identify high-risk patients appropriate for SICs, for which oncologists receive behavioral nudges.^41^ Using this approach, the rate of SICs was significantly higher (13.5% vs 3.4% without intervention), and use of anticancer therapy within 14 days of death was reduced; there was no effect on other EOL quality metrics.^41^ Though notable, there is opportunity to further increase rate of SICs among patients. Furthermore, patients identified for behavioral nudges may not be aware they are high-risk, and clinicians may not understand or be able to explain why a patient is deemed high-risk.^42^

It is challenging to communicate how predictive models work. Lack of explainability may impact clinician trust and acceptance of CDS systems based on AI.^9,12^,^43–45^ The European Union now mandates a right to explanation of all decisions made by automated or AI algorithmic systems,^46^ new US Food and Drug Administration regulations reflect a similar requirement,^47^ and recent advocacy links ethical deployment to appropriate trust.^7^ Explainability allows an end-user (ie, clinician) to understand how a model works and rationalize a specific outcome.^44^^,^^48^ Clinicians must understand the reasoning behind AI-based output when deciding to accept or reject AI-based recommendations.^43^^,^^49^ While required, explainability is not sufficient for clinical use. AI-based CDS systems should also be interpretable to support end-user implementation in a given context.^50^^,^^51^ When clinicians and patients may be influenced by the output of models, they deserve an accounting of how a decision is made,^52^ how it is verified, and how it should be interpreted in their context, delivered through a usable system that supports workflow and patient care.

Our team identified a need to support communication about predicted survival while oncologists and patients consider outpatient treatment decisions. Our overarching goal was to design a CDS tool to help oncologists identify patients with advanced solid tumors (eg, brain or nervous system cancer, or any other solid tumor with metastases) who are at the disease trajectory “tipping point” when continuing treatment is unlikely to extend life, then support conversations about prognosis and next steps. The tool uses a validated ML model described elsewhere^37^^,^^53^ to assess 6-month survival and a graphical user interface to communicate model output and recommendations. The purpose of this manuscript is to describe: (1) our user-centered process for designing an interface intended to be explainable and interpretable from a clinician perspective, (2) generalizable design decisions and resulting interfaces, and (3) feedback from oncologists to guide improvements and next steps. Findings address a noted gap in clinician involvement^54^ and user studies exploring clinician needs^44^ while developing AI-based CDS.

## Methods

### Design approach

**Figure 1.**
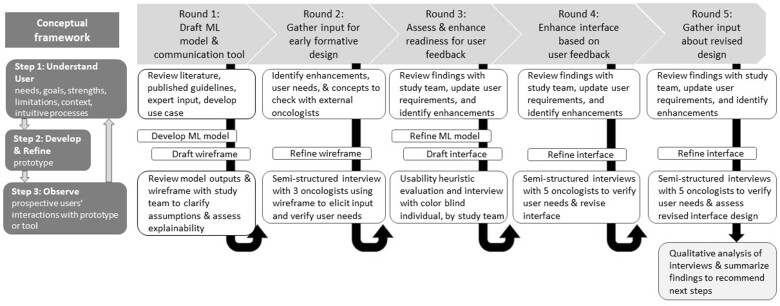
User-centered process for designing a prognostic model and interface to communicate 6-month chance of survival. The conceptual framework is based on a graphical depiction of user-centered design described by Witteman et al. User-centeredness increases occur between Step 1: Understand User and Step 2: Develop/Refine. Prototype fidelity increases occur between Step 2: Develop/Refine and Step 3: Observe phases. Knowledge increases occurs between Step 3: Observe and Step 1: Understand User during the next iteration.

To design an interface that communicates prognosis, we adapted an iterative design conceptual framework^55^ based on seminal Human Factors research (see Figure 1), and applied user-centered design principles,^56^^,^^57^ particularly adapting Nielsen’s usability heuristics^58^ for usability assessment and qualitative analysis. The conceptual framework^55^ involves rounds of iterative design, each including 3 steps. First, to establish or increase user-centeredness, one must understand users’ needs, goals, strengths, limitations, context of use, and intuitive processes.^55^ Second, to establish or increase prototype fidelity, an ML model or interface is developed or refined based on the use case and synthesis of findings from prior rounds. Third, to increase knowledge about user’s needs, prospective users should be observed interacting with the prototype. We considered metrics from the ELICIT framework^59^ to understand user requirements about the predictive model, interface usability (ease of use, understanding), and satisfaction (perceived usefulness, trust). To assess usability, we adapted Nielsen’s heuristics by adding a Trust and transparency heuristic.^60^

We engaged an interdisciplinary team involving clinicians from the Huntsman Cancer Institute throughout design. Study team members provided expertise in informatics (C.J.S., K.K., and M.G.N.), data science (G.C. and M.G.N.), human factors and qualitative methods (T.T., C.H.S., and J.-W.G.), and oncology care including a medical oncologist with over 20 years of supportive and palliative care experience (A.C.B.), and an oncology clinical pharmacist (J.P.M.) with 8 years of cancer care experience. Oncologists outside the study team participated during 3 rounds.

The University of Utah’s Institutional Review Board approved all research described.

### Iterative design with formative assessment

**Figure 2.**
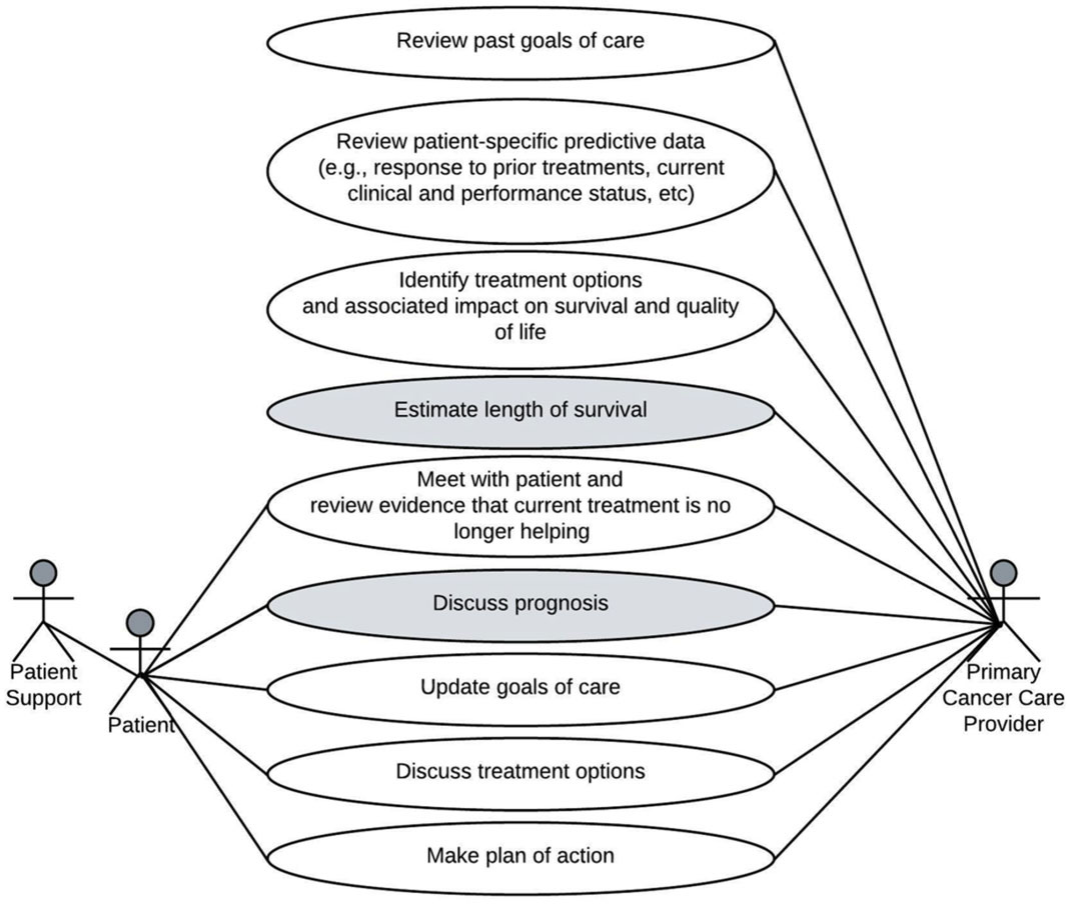
Use case for prognostication when making treatment decisions during outpatient care for advanced solid tumors. This use case describes actors and tasks for the situation when estimating survival is relevant for patients with advanced solid tumors who are considering a new line of treatment during an outpatient clinic visit. Shaded tasks involve prognostication.

During round 1, we defined a use case focused on outpatient care of patients with advanced solid tumors and 6-month survival prediction (Figure 2). The use case^61^ considered SIC guidance,^31^ nationally endorsed quality metrics,^21^^,^^22^^,^^62^^,^^63^ and clinical expert opinion (A.C.B. and J.P.M.). Based on our use case, we developed a ML model to predict 6-month survival among patients with advanced solid tumors,^37^ and drafted a low-fidelity wireframe using Microsoft PowerPoint focused on model inputs and outputs. The goal was to explain the model and elicit team feedback, particularly concerning face validity, clarity, and usability during clinical care. During team meetings, grounded by the initial design, we clarified user needs and differing assumptions expressed by clinical and technical experts.

Subsequent rounds spanning 15 months involved: (1) summarizing feedback to clarify user needs and required enhancements after reaching team consensus during biweekly meetings and through email, (2) refining the model and/or interface, and (3) seeking user-centered feedback through formative evaluations. During round 2, we modified the interface and performed semi-structured interviews with 3 oncologists. During round 3, the model was enhanced and we used Sketch software to create an advanced interface design. To assess appropriateness of colors, we sought input from an adult with red-green color blindness, and reviewed guidance for color blind audiences.^64^ To assess usability, we performed an internal expert review based on usability heuristics. Two usability experts (authors T.T. and C.H.S.) independently assessed the interface using a checklist based on Nielsen’s design heuristics adapted to also assess Trust and transparency.^58^^,^^60^ The checklist and instructions are based on prior research^60^ and included in Supplementary Material (Heuristic Evaluation). After independent assessment, the experts established consensus and recommended enhancements based on observed violations. During round 4, the interface was enhanced to fix obvious problems after 2 of the 5 interviews. During round 5, the interface was enhanced and the revised version was evaluated by 5 oncologists.

Throughout design, we were unaware of an existing interface for reference or comparison that addressed our use case.

### Oncologist feedback

#### Recruitment

We recruited oncologists from Huntsman Cancer Institute (HCI) specialized in breast, gastrointestinal, lung, or genitourinary cancer, as these are commonly occurring cancer types in the United States.^65^ We staged invitations to balance gender and specialty and included 2-5 users per round. We aimed for at least 10 users and 3 rounds, as most usability issues are identified with as few as 10 users.^58^ Participants received a $100 gift card. Thirteen oncologists participated, including specialists in cancer of the breast (*n* = 5), gastrointestinal system (*n* = 4), prostate (*n* = 2), and lung (*n* = 2), averaging 7.8 years of practice (SD= 8.4, range = 0.25-30); 7 (54%) were male. In each round of user testing, participants represented 3 or more different specialties.

#### Vignette and interface development

To create vignettes and interfaces for feedback, we leveraged de-identified patient records used during model development.^37^ We identified patients with advanced breast, lung, prostate, or colon cancer with ≥3 lines of therapy (LoT) for anticancer treatment and a variety of outcomes 6 months after starting a new LoT, enabling participants to see progression across LoT in the same patient. Our process for defining LoTs (eg, anticancer therapy entered into EHR treatment plans, including chemotherapy, biologics, targeted therapy, immunotherapy, or hormonal therapy) is described elsewhere.^37^ We populated interfaces with representative patient data and predicted survival for each treatment decision point when a new LoT was started. Our clinical experts (A.C.B. and J.P.M.) defined vignettes representing a patient’s journey. For each case vignette, a series of interfaces corresponding with LoTs were generated.

#### Interview script development

A multi-part semi-structured interview script was designed to address Framework Step 1, gathering qualitative data to understand user-needs, goals, strengths, limitations, context, intuitive processes; clarify information needs in the context of the interface presented; and identify usability problems and helpful features of the presented interface.

The script included an introduction, followed by prompts and questions for a critical incident interview^66^ to understand participants’ behaviors and information needs when assessing and communicating prognosis to a patient with advanced cancer and 6‐12 months of expected survival. Next, a cognitive walk through and contextual inquiry was used to elicit feedback and discover user misconceptions and mismatch between interface design and user mental model or workflow. Using a think-aloud protocol,^67^ participants were asked to describe what they were thinking and understood, first focusing on the entire interface and then on each section within the interface. Participants were prompted to talk about what they saw, what it meant, what they could do with it, what they liked or didn’t like, or anything that was confusing. Next, a series of open-ended questions further explored oncologists’ perceptions about utility, clarity, trust and impact, and elicited recommendations and other comments. These questions were directly linked to usability constructs with the interface presented and prompted users to expand on earlier comments. Next, a set of Likert-style rating questions, using a 7-point Likert scale (agreement ranged from 1-low to 7-high), addressed usefulness, trust, agreement with recommendations, confidence and intention to use, likelihood of recommending to a colleague, support for awareness of patient prognosis, and alignment with expectations. The questions were developed based on objectives of the study. Finally, 3 questions ascertained years in practice, specialty, and gender identity. All questions were designed to probe for feedback, guide design, and were tightly aligned with CDS tool goals and good design practices (eg, checking match and clarity).

The script and questions were assessed initially for face validity with the study team and pilot tested. The script used during the final 10 interviews is available in Supplementary Material (eMethods), and is similar to that used during the initial 3 interviews.

#### Data collection

Interviews were performed remotely and audio recorded using Zoom. After consenting, 1-h semi-structured interviews were performed (by T.T. and C.H.S.) following the interview scripts. Up to 4 case vignettes (including a simulated patient story and associated interfaces) were presented for the think-aloud protocol with contextual inquiry. Non-interactive interfaces were displayed on a shared screen controlled by researchers. Audio recordings were stored in a secure environment, professionally transcribed, and de-identified.

#### Data analysis for incremental enhancement

Transcripts from rounds 2 and 4 were used (by T.T. and C.H.S.) to summarize and present findings during team meetings. Findings focused on user’s goals and tasks and information needs derived from the critical incident interview, as well as feedback about missing content or the usability of included content. Specific enhancements derived from an issue were then generalized to identify design decisions that should be applied throughout the current interface and considered when future issues are discussed and enhancements are implemented. Documentation of design decisions and interface specifications were continually updated. After establishing all design decisions, the decisions were mapped to usability heuristics to illustrate the scope and change in issues as users responded to more advanced designs.

For the final 10 interviews, we calculated an overall Likert score for each participant by averaging all responses.

#### Qualitative analysis

After completing round 5, all transcripts underwent qualitative analysis to further describe user’s perceptions about usability and satisfaction, and assess whether concerns identified during rounds 1 through 4 were fixed or recognized for future enhancement. Qualitative analysis of transcripts was performed by trained qualitative researchers (C.H.S. and J.-W.G.) using NVIVO (QSR International) software. A directed content analysis was performed based on 11 usability heuristics adapted for CDS, including Nielsen’s 10 usability domains^58^ and an additional heuristic of Trust and transparency.^60^ We analyzed sections of the interview transcripts containing the cognitive walk through and contextual inquiry, and follow‐up questions. A codebook, organized by usability domains, focused on positive perspectives, usability issues, and missing items as shared by oncologist participants (see eMethods in Supplementary Material). The unit of analysis was a sentence or paragraph, which could be assigned to more than one code.

Inter-rater reliability (Cohen’s Kappa coefficient) was calculated using NVivo. Researchers independently coded one same transcript based on 11 usability heuristic domains.^58^^,^^60^ The average Cohen’s *k* was 0.85 (ranging from 0.53 to 1.00). Six usability heuristic domains were used during content analysis. Consensus was reached after a meeting among coders to discuss discrepancies, resolve disagreements, and revise the codebook. Researchers independently coded remaining transcripts, then reviewed and revised coding together. Exemplar quotes were used to highlight key findings.

## Results

### Design decisions and outcome

**Figure 3.**
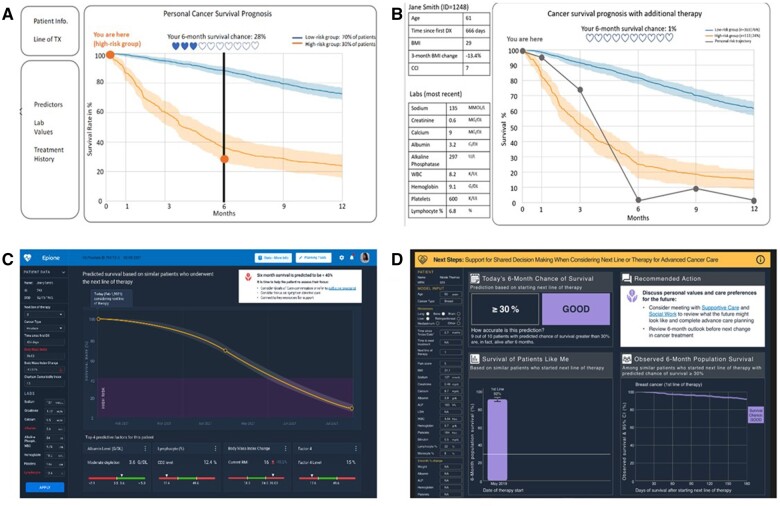
Example of interim designs generated during user-centered design process. (A) Presents the initial wireframe used in round 1. (B) Presents the refined wireframe used in round 2. (C and D) Present the 2 versions of the interface used in round 4. Mock patient data were used for all visuals. The image should be displayed in color to visualize as intended.

Interfaces used during rounds 1 and 2 provided a common visual and terminology for team discussions and clinician input, and were useful to determine what content to present, clarify model function, and establish an appropriate threshold for classifying survival risk. Importantly, they exposed misassumptions among clinical and technical experts, such as initial beliefs that the model output was expected survival rather than a value to classify risk. The discussions triggered questions and the presentation of additional information by technical experts (described elsewhere^37^), so that study team clinicians could assess clinical relevance, features, and the gold standard mortality data, and advise about data pre-processing, both of which are important categories of clinician involvement for AI system design.^54^ Based on findings during rounds 1 and 2, we applied design decisions to enhance the model and interface that mapped to Trust and transparency and Match between the system and real world (Table 1). The progression of interim interfaces is shown in Figure 3.

**Table 1.** Key generalizable design decisions impacting early interface design, including example rationale, grouped by relevant usability domain.

Design decision		How and why decision was applied	Usability domain
No.	Description		
1	Display data for *all* features included in predictive model	Initially, we only presented the 14 most predictive features based on SHAP scores. We expanded to include all 45 features because this section addresses the question: What data or logic were used for prediction?	Trust and transparency
2	Update predictive model to include universally expected features despite low prognostic value	Initially, we only included features that were clinically relevant, available in the EHR, and predictive based on SHAP scores (feature selection is described elsewhere^37^). Based on user requests and expectations, we added cancer type and sites of metastasis to the predictive model even though they had very low SHAP scores (ie, low predictive value in model).	Match between system and the real world
3	Tailor information based on patient’s current predicted Boolean risk category for surviving 6 months	Initially, we presented survival trajectories for both risk groups in one graphic and predicted survival 1, 3, 6, 9, and 12 months in the future. After adding error bars representing prediction variance, we noted wide variation beyond 6-month predictions. We updated to focus on the population of patients with the same risk, cancer type, and line of therapy (ie, “patients like me”) and predict only the 6-month horizon.	
4	Communicate recommended actions tailored to predicted risk	Initially, no recommendations were shared. We defined logic and added a “Recommended Actions” section with information for the oncologist and patient to consider, tailoring recommendations based on the patient’s predicted 6-month chance of survival.	

In contrast, during rounds 3 and 4, the more advanced interfaces elicited input that refined what to present but also how and why we present specific information. User needs motivated additional design decisions (Table 2). For example, oncologists wanted to use the interface to “tell a story” as they discuss prognosis. They wanted content to be valid and easily interpretable, so they can be comfortable sharing and explaining it with patients. Design decisions led to revising the order of sections, and use of “low” and “likely” to express risk, matching oncologists’ worldview.

**Table 2.** Key generalizable design decisions impacting advanced interface design, including example rationale, grouped by relevant usability domain.

Design decision		How and why decision was applied	Usability domain
No.	Description		
5	Clearly indicate the appropriate context for use: purpose and *when* to use the tool	Initially, we only titled the survival graph, not the full interface. We updated the interface to include: (1) an overall title to communicate how the tool may be used and for which patient population it is relevant, and (2) an “info-button” linking to a resource page that includes “frequently asked questions” explaining terms and methods, the citation for the model validation publication, and information about development team.	Consistency and standards
6	Specify questions expected to be answered by the content presented.	Each section of the interface should address an information need. While the questions related to information needs are not visible to the user, we defined and refined the questions in design documentation which helped clarify scope for each section as enhancements were applied.	
7	Use color palette that allows any user to discern information shared using color.	We adopted a color-blind friendly palette that included colors we wanted to use (eg, green [#BDD9BF], gold [#FFC857], purple [#A997DF], and gray [#2E4052]) as well as black and white. We chose colors that would not communicate alarm: green is used for “low” chance of survival and purple for “likely” chance of survival; we specifically avoided red (#E5323B).	
8	Display information relevant for patient care decision-making, not decisions made by the ML algorithm	Initially, in graphics, we displayed the belief threshold used by the model (30%) to assign patients to the Boolean risk category of “low” vs “likely” to survive 6 months. This value was erroneously interpreted to be the patient’s expected likelihood of survival. After reviewing visualizations derived by the technical team, this error was identified and corrected. Instead, we now present observed survival rates that were calculated using Monte-Carlo simulations for patients of the same risk category, cancer type, and line of therapy (ie, patients like me)	Aesthetic and minimalist design
9	Use clear language to help clinician explain content (eg, titles, labels)	Orient text so it is from the patient’s perspective. Attempt to ensure all text is accessible for expected users, including patients with whom oncologists may share the interface (ie, fifth grade reading level).	
10	Remove unnecessary information	We incrementally removed text and graphics that did not add value in addressing a specific user information need.	
11	Illustrate uncertainty	To communicate uncertainty around predicted survival, we present confidence intervals in the survival curve, and error bars in the bar chart. Calculation of confidence intervals is described elsewhere.^37^	Trust and transparency
12	Remove confusing information	Initially, we included the model output value used to assign risk (see Figure 3: hearts and personal risk trajectory). In later interfaces, we included text and lines referencing the 30% threshold value used by the model to classify risk. Users shared: “Honestly, I have no idea why anyone just arbitrarily picked 30 percent as being good … that number should be higher” (#5). However, these 2 values are model artifacts not relevant for clinical decision-making. So, we removed references to values used by the model to classify risk.	
13	Use terminology that matches the oncologist’ world view.	Initially, we used “low” and “high” risk [of mortality] on a survival % graphic, creating a cognitive disconnect because “low” risk meant “high” survival. This issue was partly resolved when we tailored the interface by risk status. We then used the terms “low” and “good” to communicate 6-month of survival chance, but “good” was not acceptable: “good is subjective…I think what’s good from an oncologist’s perspective is very different [from] a patient perspective.” (#6) We then updated terms to “low” and “likely” which matched user’s expectations: “How am I going to say this? We think it’s likely that you’ll be alive at six months.” (#7)	Match between system and the real world
14	Include information that meets an oncologist’s needs when communicating prognosis in context of treatment decisions.	We considered information needs and components of a prognosis discussion shared by oncologists during interviews. We purposefully articulated the questions addressed by each section. One participant noted: “What I really like about graphs like in the bottom right, is it completely removes any external contextualization and it just allows the patient to see the information, to absorb it and to decide for themselves what the qualitative term is going to be…does this look…favorable for me or not? Now that being said, I do like having some type of information as is presented [on] top…because it’s very straightforward and it’s very intuitive…I find that [displays] like this that include both of that information tend to be the most helpful for the patients.” (#5)	
15	Arrange sections in logical order to “tell the story” and discuss prognosis and next steps with a patient.	We arranged sections to support the user to start at the top left section of the interface and then move to the right. We placed the most important sections across the top, including key information about risk and recommended actions. Supplementary Material about input values, uncertainty and trends are along the bottom. The order matches the flow of reading in English (left to right) and with information shared by an expert with extensive experience communicating prognosis with a patient with advanced solid tumor.	
16	Communicate context	To support user’s needs to “tell the patient’s story” and share trends over time, we added historical information about predicted risk when starting prior lines of therapy. Future enhancements could display trends for input or other values (eg, albumin and weight)	

#x, refers to interview number.

**Figure 4.**
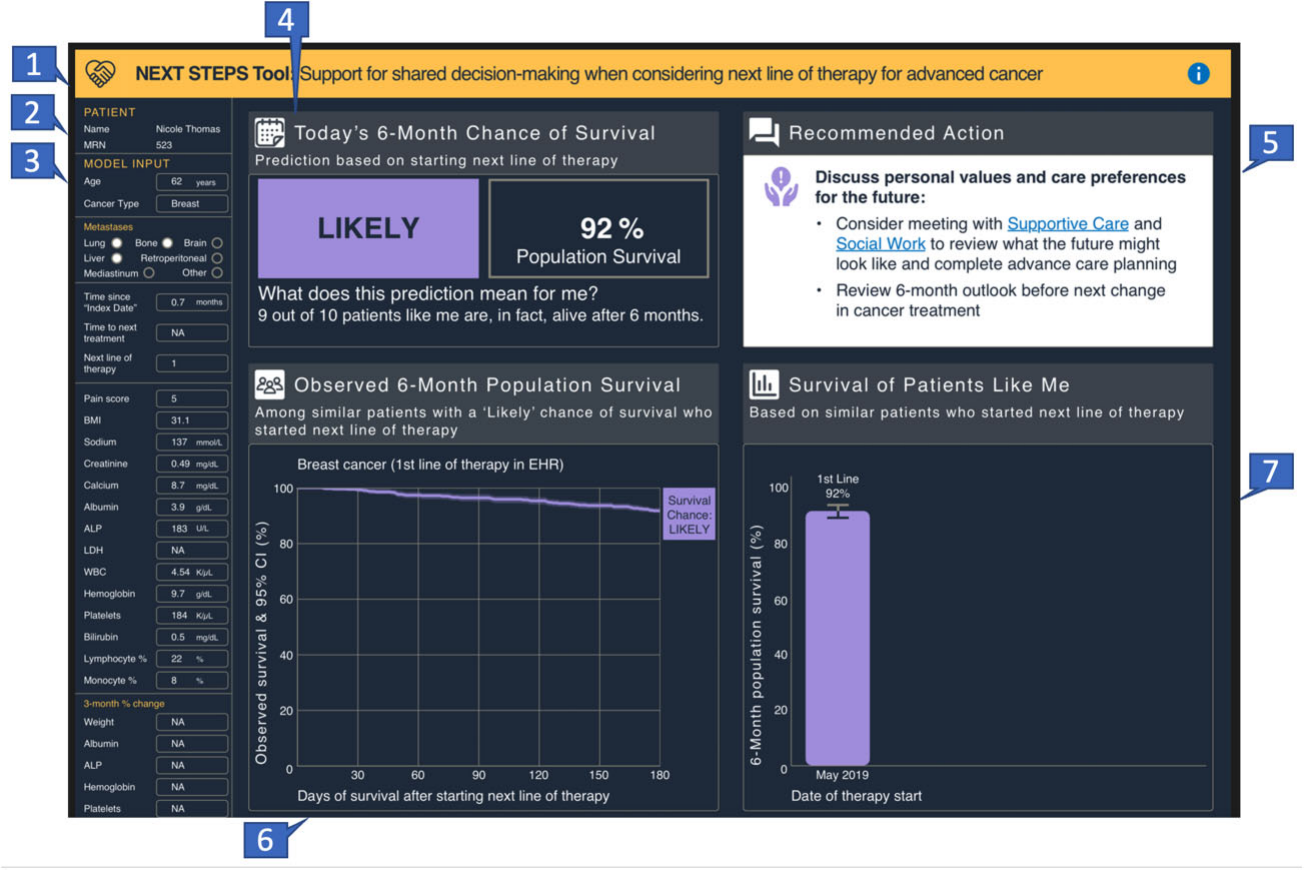
Final interface design indicating discrete sections and illustrating information presented when a patient had a likely chance of surviving 6 months. Illustrates a mock patient’s chance of survival when considering a first line of therapy. Recommended actions are based on a likely chance of survival classification. The image should be displayed in color to visualize as intended. The numbers represent sections described in Table 3.

**Figure 5.**
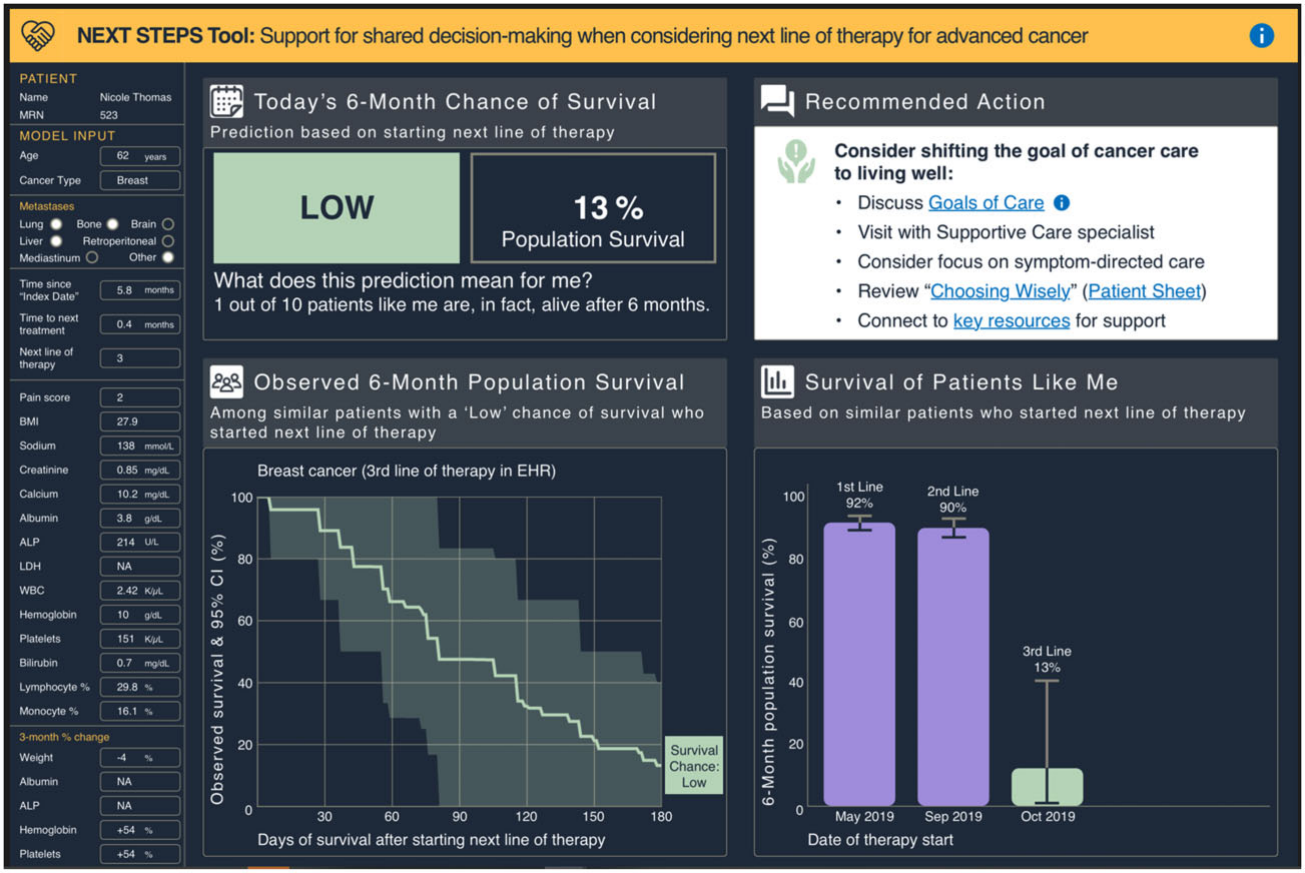
Final interface design illustrating a mock patient’s progression from likely to low chance of surviving 6 months. Illustrates a mock patient’s chance of survival when considering a third line of therapy and shows progression from likely to low chance of survival after the first, second, and third lines of therapy. Recommended actions change based on chance of survival classification, and this view is based on a low chance of survival. The image should be displayed in color to visualize as intended.

The design process resulted in an interface with 7 sections (Figure 4). Each section answered user-focused questions (Table 3), and would be updated using recent information when making a treatment decision and considering a new LoT. Over time, a patient’s predicted 6-month survival changes. Figure 4 presents information based on the predicted 6-month survival when considering a first line of anticancer therapy, when chance of survival was likely. Figure 5 presents predicted 6-month survival when considering a third LoT, when the chance of surviving 6 months was predicted to be low. The design specification corresponding with this final version is available as a Supplementary Material (Design Specifications).

**Table 3.** Description of sections within the interface.

Section number	Purpose of the section	Questions addressed by content in the section
1	Banner with title and info button which links to system description and references	What is the purpose of this tool? Where do I go for more information?
2	Identifiers	Which patient is the focus of this information?
3	Inputs	What data (features) were used to make the prediction?
4	Key findings	What is my 6-month chance of survival – “likely” or “low”? What was the 6-month survival among patients like me (population with similar risk status, line of therapy, and cancer type)? What does this prediction mean from patient’s perspective?
5	Recommended actions and EHR links, depending on risk	What are recommended actions? How do I implement these recommendations?
6	Expanded data for “similar patients”	How much uncertainty is there in survival among patients like me?
7	Prognosis history	Has my predicted status and population survival rates changed since making prior treatment decisions?

### User feedback

Qualitative analysis of transcripts revealed that most (91%) participants’ comments concerned 4 usability domains (Recognition rather than recall, Match between the system and the real world, Trust and transparency, and Aesthetic and minimalist design) which are defined in Table 4. Feedback concerning Consistency and standards and Help and documentation was uncommon. Table 4 includes exemplar feedback of both positive statements and issues expressed. As noted in Table 4, some issues identified early in the design process were resolved, while others were not. Three key themes concerning needed enhancements emerged: (1) revise graphic labels (eg, use months, not days); (2) include additional information in the model and/or interface (eg, performance status, external LoT, symptoms); and (3) add functionality to support workflow (eg, allow manual entry of performance status or external LoT; automate referrals for social work, supportive, or hospice care; and embed the system within the EHR workspace).

**Table 4.** Example feedback from oncologists about the interface, by round and usability heuristic domain.

Usability domain	Rounds 2 and 4 (*n* = 8 participants)	Round 5 (*n* = 5 participants)
Match between the system and the real world (Definition: The system should use words, phrases and concepts familiar to the user, rather than system-oriented terms. Follow real-world conventions, making information appear in a natural and logical order)		
Positive comment	“As oncologists we sometimes have rose-colored glasses on and like to overestimate the benefits of second, third, fourth line treatment. And so, I think that this can maybe ground you and bring you back to reality like, hey, look it’s probably not such a good idea. Let’s think about alternatives.” (#7) “I think this would be useful for family members.” (#3) “…helpful in…patients who…see cancer as like a battle that they have to fight…often unwilling to stop treatment despite all evidence that treatment might harm them rather than help them.” (#7)	“The tool [is] communicating the uncertainty…in a way that I’m able to interpret that and maybe communicate to the patient.” (#9) “It will give me more confidence that there’s a good survival and we can be aggressive.” (#10) “It would [help] me be [more] quantitative about what I should expect for the patient and help the patient understand that.” (#11) “It would be particularly useful…for patients who are wanting to push on despite me feeling that they’ve maybe used up their good options and would be better served with hospice care.” (#11) “Low makes sense…I can understand likely as well. So, low to me, I think it’s perfect for this point, it’s less than 50 percent.” (#9)
Issue	“Often…what I’m trying to convey is your survival chance with and without treatment. There’s no without treatment on this graph.” (#2)	“Tool needs to be used with someone to explain it for patients” (#13) “Often times we are getting new patients…who have been formally treated in community…[When] we think of their first treatment at Huntsman, it’s probably their second or third… The system [should be] able to adjust for that fact.” (#9)
Trust and transparency (Definition: Trust in the system should be supported by transparency and disclosure of relevant information)		
Positive comment	“If it was a validated tool…I would have a high trust in it.” (#5) “I wholeheartedly agree with the recommended actions.” (#7)	“They look valid…The error bars and the confidence intervals…makes it clearer that some of the data are fuzzy.” (#10)
Issue	“EHR data isn’t always accurate…the machine learning [algorithm] can be coded inaccurately…a quality control step to make sure that the input into this model is as accurate as possible would be important.” (#5)	“I just need the literature behind how you developed the tool.” (#12) “If they’ve had three or four lines of therapy somewhere else and then they get the fifth line of therapy here even though it’s the first one here they’re going to have a very different prognosis than somebody who has been treated here the whole time.” (#11)
Aesthetic and minimalist design (Definition: Every extra unit of information in a dialogue competes with relevant units of information and diminishes their relative visibility.)		
Positive comment	“That [holding hands] icon is nice. …[It] obviously represent[s] …we’re coming to a decision together…That’s a nice way of…displaying that language in graphic form.” (#7)	“I like the tool…It captures key information…This graphic [showing likelihood of surviving beyond six months] helps hit that quickly.” (#9)
Issue	“Alot of people have trouble with graphs…particularly a graph that’s got three lines and any two of which have error bars.” (#2) “I would do it with a single curve that…best fit the patient.” (#1) (NOTE: issue resolved)	“I feel like it doesn’t…necessarily communicate to someone of a low education status.” (#9)
Recognition rather than recall (Definition: Minimize the user’s memory load by making objects, actions, and options visible. Users should not have to remember information from one part of the process to another. Instructions for use of the system should be visible or easily retrievable)		
Positive comment	“This figure makes sense.” (#4)	“If this pops up as a recommended action, I can see that as being a useful communication tool.” (#9)
Issue	“Trends are really important…Albumin is probably the most important… I look at weight…knowing what it’s trended…is really important” (#2)	“…the ECOG, the performance status is missing, which is something that you need to decide for further treatments.” (#12) “What are their goals of treatment?” (#12)
Consistency and standards (Definition: Users should not have to wonder whether different words, situations, or actions mean the same thing. Standards and conventions in product design should be followed.		
Issue	“When you say ‘Next steps,’ I’m expecting very concrete information. That doesn’t provide any concrete information.” (#5) (NOTE: issue resolved)	“…on the X axis the number of days, instead of six months, so they are different metrics… Someone might have to do mental math to convert 180 days to six months…use months to keep it simple.” (#9)
Help and documentation (Definition: While it is better if the system can be used without documentation, it may be necessary to provide help and documentation. Such information should be easy to search, focused on the user’s task, list concrete steps to be carried out, and not be too large)		
Issue	“I don’t know [what] this Frequently Asked Questions will…show the provider…Maybe you can just put up a note what index date means or you can put something…the provider…can search.” (#4) (NOTE: issue resolved)	“This tool needs to be used with someone to explain it for the patient…especially if…you show a very low survival.” (#13)

Text in [] provides additional information to enhance quote clarity. (#x), interview number. We do not include positive comments about “Consistency and standards” and “Help and documentation” because this category was uncommon for both positive comments and issues, and positive statements are not expected when the system functions as expected.

The type and quality of qualitative feedback evolved as the interface advanced. During rounds 2 and 4, participants indicated the tool would help patients; but during round 5, participants indicated the tool could also help oncologists make decisions and communicate prognosis with patients. One oncologist noted it would be “useful anytime [I’m]… having a prognostic discussion with the patient, particularly when prognosis is poor or changing.” Another oncologist shared, “It will give me more confidence that there’s a good survival and we can be aggressive.”

In contrast, Likert score rankings of follow-up questions did not change as the interface advanced. While one respondent reported the interface would not help with prognosis awareness as they already know their patient’s prognosis (and gave a score of 1), all other responses were neutral or positive and there was no significant difference in overall rankings between round 4 (*n* = 5; mean 5.8; SD 0.7; range 3-7) and round 5 (*n* = 4; mean 5.6; SD 0.6; range 1-7). No new design recommendations were generated; however, overall positive scores supported continued effort to develop a tool to communicate ML-based prognosis.

## Discussion

Developing trust in AI for integration into health care workflows requires clinical input during design.^54^ In our study, we used iterative feedback from clinicians to design a visual tool to support discussions when considering a new treatment for advanced solid tumors, and identified interface content that may improve trust in AI-based prognosis applications. Oncologists want an intuitive and trustworthy AI tool that matches their ways of explaining prognosis and next steps, particularly when having SICs. They see value in automated prognostication, sharing objective information, and articulating next steps in an interface. Our design process led to an unexpected finding. When presented with a visual, oncologists preferred display content for interpretability over explainability to better support treatment discussions with patients and caregivers.

### Design process

Here, we describe a replicable design process that addresses a gap in the literature concerning user studies for understanding clinician needs when designing ML-based CDS^44^^,^^54^ and aligns with recently published recommendations.^15^^,^^44^^,^^51^^,^^68^ Grounded by well-established user-centered design principles,^55–57^ we clarified the context for implementing AI through a use case and user input, and sought clinician involvement early and throughout system design even though involvement is typically limited to the start or end of ML-based design projects.^54^ The need to improve user-centered design of EHR applications is well documented,^69^^,^^70^ even for well-established tasks (eg, ordering X-rays). Use of AI-generated information within clinical workflows is new, and the tasks, goals, and information needs associated with its use are less understood. Thus, user-centered design techniques are especially critical to ensure AI-related systems are designed to meet a proposed use case and remain usable and safe after implementation.

Visuals were particularly valuable for grounding discussions, providing a springboard for brainstorming, shared understanding and language, and imagining something new among team members with varying AI expertise. As recommended,^51^ we found that early use of a wireframe was efficient for increasing understanding about how the model worked and exposing model limitations and misassumptions. Consequently, the underlying model^37^ and strategy for communicating risk based on model output were revised. Ongoing use of visuals and feedback loops between technical and clinical experts^43^^,^^51^ enhanced understanding of how the system worked and how clinicians need it to work, and elicited actionable feedback from clinical experts within and outside the study team. The process resulted in an interface that supports clinical use of AI by leveraging 2 recommended strategies to bridge clinician needs and developer goals: contextualize model output,^48^ and enable holistic patient assessment by providing context and cohort-level information.^68^

### Design outcome

Design decisions reflected usability best practices to share content that answers user-focused questions articulated by clinicians. The need to know the prediction output and what data are used to make a prediction is not surprising, and noted elsewhere.^44^^,^^48^ However, unlike the focus on explainability reported by others in settings where CDS supports urgent decision-making by clinicians,^43^^,^^48^^,^^50^^,^^68^ our process resulted in a design that prioritized interpretability over explainability. Most notably, clinicians wanted patient-specific context to be able to interpret the information and feel comfortable sharing and explaining it to patients, an important attribute as identified by Vellido.^51^ Elements that supported interpretability and would help the clinician to “tell a story” when interacting with a patient included a “local” view using the patient’s predicted output (ie, low or likely chance of survival and patient-specific recommended actions), a graphic to show “where the patient has been,” a “global” view using population data describing survival patterns among similar “patients like me” (cancer, LoT, and risk category), and use of confidence intervals to illustrate uncertainty. Requirements for interpretability to support end-user implementation in a given context are not well-described in the literature.

Positive user feedback supports the belief that a tool for communicating prognosis when considering new treatments has value for oncologists. In a similar study, when queried about the value of a hypothetical predictive tool without visualization to describe a tool, oncologists raised many concerns about accuracy, biases, and ethics.^71^ Sharing visualizations as part of our design and feedback strategy likely enhanced clinician understanding about a plausible clinical context for using an ML-based tool^68^ and allowed us to identify actionable usability heuristic violations^58^^,^^60^ that were either addressed or defined for further investigation before real-world testing. Additionally, findings indicated readiness for formative testing and design with another important set of users whose perspectives are not well described in the literature: patients and their caregivers.

### Limitations

Our research has limitations, particularly 2 each concerning methods or outcomes. First, formal content analysis of interview scripts was not performed between rounds and prior to interface enhancement, delaying recognition of selected usability issues. Second, our findings may not generalize to other sites, such as those with more diverse patient populations. Third, while the interface has value when considering a new LoT, it does not currently include the alternative when no treatment is sought. Given this gap, the interface does not currently meet criteria for shared decision-making^72^ or patient decision aids.^73^ Even so, tool features align with essential inputs for clinical decision-making, which include research evidence, clinical expertise, and patient preferences and values.^74^ Finally, we added 2 features to the ML model (cancer type and sites of metastases) in response to user expectations that these features are important, despite prior analysis of SHapley Additive exPlanations (SHAP) scores indicating they had minimal impact on prediction performance.^37^ We did not further explore the validity of this expectation, missing an opportunity to contribute to medical knowledge and better understand the relationship between sites of cancer and survival after a patient meets criteria for having an advanced solid tumor.^44^ Despite limitations, our study demonstrated how a formative user-centered design approach may be used to design a well-accepted system prior to *in situ* implementation.

### Future directions and implementation considerations

This study represents the beginning stages of interface development for a CDS prognosis tool, and exposes unique implementation considerations that will require further development and real-world, summative evaluation.^75^ First, the clinician requirements for supporting explainability in our context (prognosis and outpatient referral for a SIC) differ from requirements reported for AI-based CDS tools that diagnose or predict an impending problem (eg, hypoxemia or delayed cerebral ischemia while receiving anesthesia or intensive care, respectively) requiring rapid decision-making.^43^^,^^68^ In our scenario, the requirements for explainability did not emerge as a first priority, and may be handled using tool-specific documentation available when needed, such as a proposed Oncology AI Fact Sheet for Cancer Clinicians.^17^ Future assessments should compare the value of documentation^17^ versus embedding explanations within a tool^44^ for different AI tasks and settings. Second, future evaluations should assess the needs for revalidation and communication about accuracy and model performance, given that AI models learn and change over time, creating a challenge highly relevant for AI-based CDS. Third, technical implementation of the tool will require unique dynamic data and knowledge management involving a knowledgebase with past person-specific prognostications and continually updated population-level outcomes for similar patients receiving care at our health system. The knowledgebase will need to be implemented before the interface can move beyond a proof of concept. Finally, our tool is intended to be implemented in the context of communication between providers, patients, and caregivers, as decisions about anticancer therapy are nuanced and many factors (eg, availability of clinical trials and model performance) must be considered. The predictive algorithm is meant to supplement clinical decision-making, and is not intended for independent decisions about treatment selection, authorization, or reimbursement. Policies and technical solutions should enable AI-based CDS systems to respectfully and transparently support users (including clinicians, as well as patients and caregivers) to appropriately interpret outputs and recommendations.^19^

## Conclusion

User-centered design, ongoing clinical input, and early use of a visual interface to communicate AI processes and outcomes are crucial for designing AI-enabled CDS tools. The interface designed using this interdisciplinary user-centered approach can communicate risk and support oncologists and patients when making treatment decisions, particularly when anticancer therapy is unlikely to extend life.

## Supplementary Material

ocad201_Supplementary_Data

## Data Availability

The data underlying this article will be shared on reasonable request to the corresponding author.
