# Supplementary material for: Design of an interface to communicate artificial intelligence-based prognosis for patients with advanced solid tumors: a user-centered approach
Source: J Am Med Inform Assoc. 2023 Oct 17;31(1):174–87. doi: 10.1093/jamia/ocad201 (PMC10746322; doi:10.1093/jamia/ocad201)
Supplement: ocad201_Supplementary_Data [file ocad201_supplementary_data.zip › ocad201_Supplementary_Data/Supplement_Design specifications document.docx]

Manuscript: **Design of an Interface to Communicate Artificial Intelligence-Based Prognosis for Patients with Advanced Solid Tumors: A User-Centered Approach**

**Supplemental file title: Design specifications**

Last updated: 2023-05-05

Compiled by Catherine Staes, PhD, MPH

NOTE: This document describes the interface used during the evaluation phase with providers and the companion patient and caregiver study. This is a document that contains work in progress and may have errors but reflects key decisions and is made available in the spirit of transparency.

Contents

[Purpose and goal 3](#_Toc134188491)

[Abbreviations 3](#_Toc134188492)

[Visualization 4](#_Toc134188493)

[Likely chance of survival 4](#_Toc134188494)

[Low chance of survival 4](#_Toc134188495)

[Q&A: Guide for interpreting the information 5](#_Toc134188496)

[General layout and design specifications 6](#_Toc134188497)

[Layout 6](#_Toc134188498)

[Specifications to be used throughout the GUI: 6](#_Toc134188499)

[Description of sections within the GUI 8](#_Toc134188500)

[Section 1. General Banner 8](#_Toc134188501)

[Section 2. Identifiers 9](#_Toc134188502)

[Section 3. Features included in the model (Left panel): 10](#_Toc134188503)

[Section 4. Key findings 12](#_Toc134188504)

[Section 5. Recommended Action, including logic 13](#_Toc134188505)

[Section 6. Graphic of Population data illustrating variation 17](#_Toc134188506)

[Section 7. Timelines of historical information 18](#_Toc134188507)

[Section 8: Information page accessed using link in header 20](#_Toc134188508)

[References 21](#_Toc134188509)

[A. Clinical Guideline 21](#_Toc134188510)

[B. Quality Measures 21](#_Toc134188511)

# Purpose and goal

To communicate:

1. expected chance of 6-month survival IF a new line of anti-cancer therapy is started,
2. survival observed among ‘similar’ patients (e.g., similar cancer type, line of therapy, and predicted risk status (low or likely)), and
3. recommended next steps so patients understand their options before making a treatment decision

 with a Goal:

- To prompt patients and providers to consider symptom-directed care when a new line of cancer-directed care is not expected to prolong life beyond 6 months.

# Abbreviations

GUI means graphical user interface

# Visualization

NOTE: final version shared with providers, ‘patients’ and ‘caregivers’ for evaluation and feedback

## Likely chance of survival


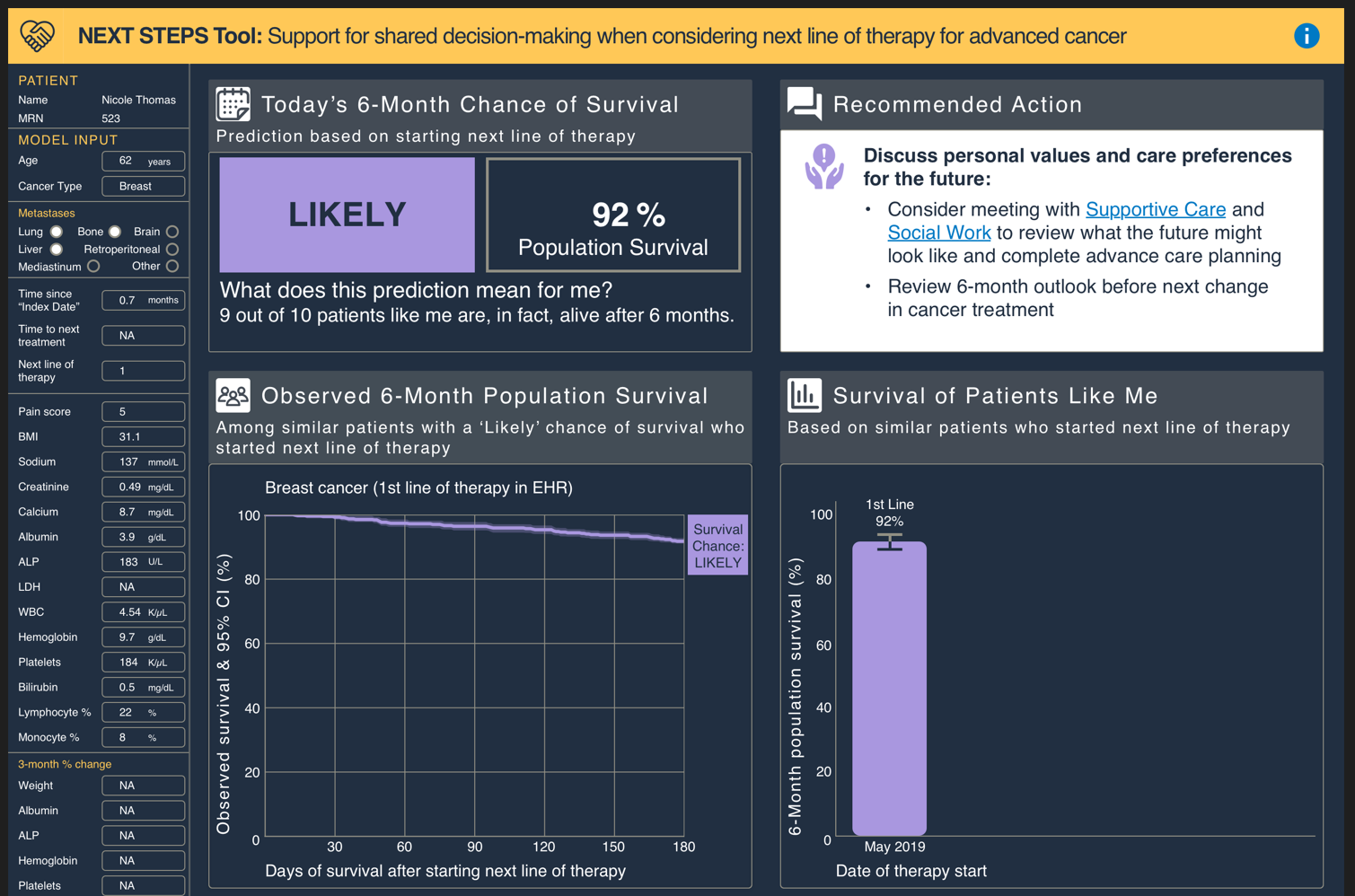


## Low chance of survival


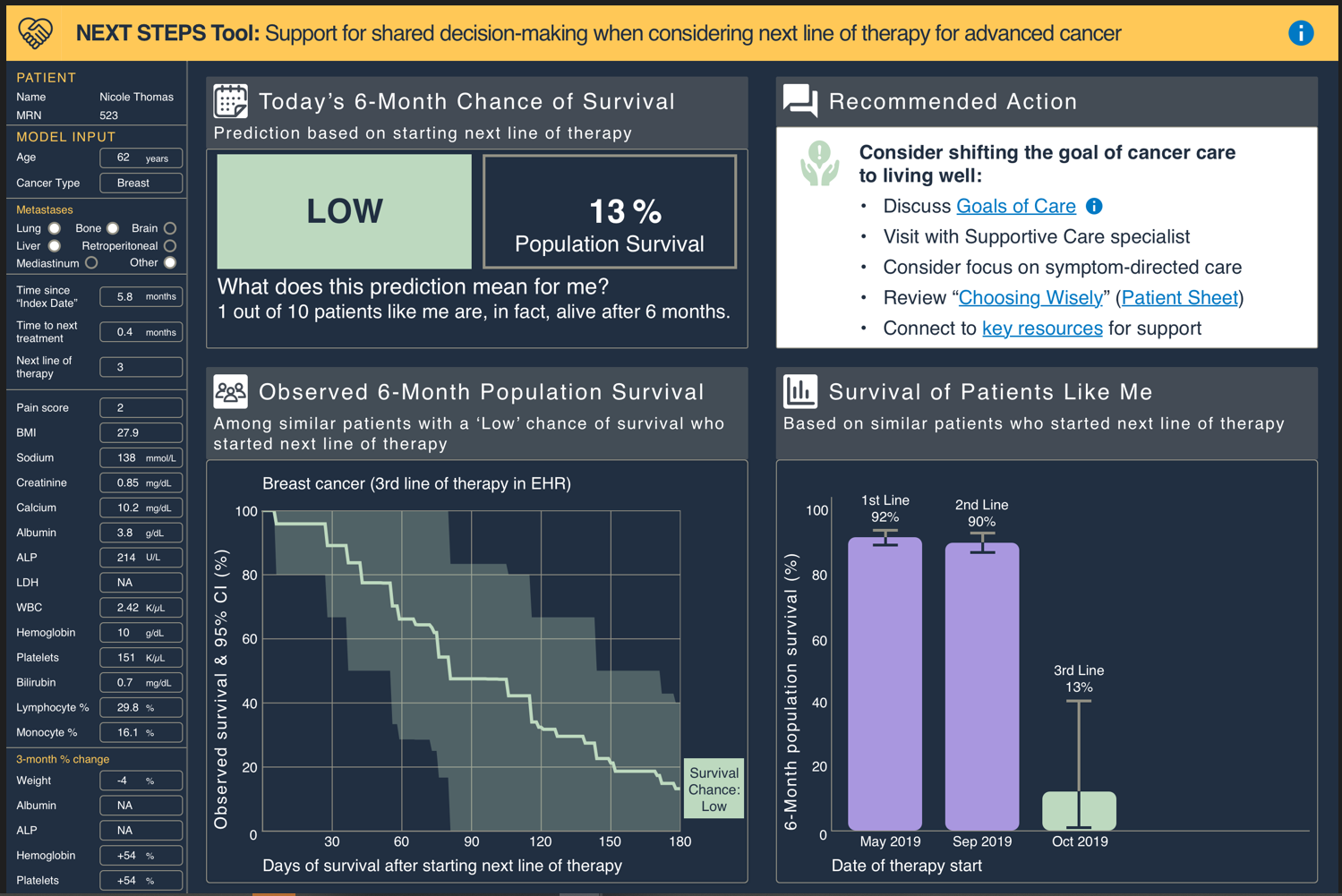


# Q&A: Guide for interpreting the information

Question 1. Which data values did you use to make the prediction?

- - Answer: the most recent data available including any data prior to the date when starting a new line of therapy. We roll-up data within 2-week intervals to summarize data in the EHR and we look back as far as 30 days if data are missing.

Question 2. What is valid to communicate about the prediction made?

- Answer: Whether the patient is above or below a threshold (i.e., 30%) used to separate those expected to be deceased or alive in 6 months. Those classified to be deceased we say have a ‘low’ chance of survival. Those classified to survive we say have a ‘likely’ chance of survival.
- NOTE: The actual value of the prediction is not a probability to share, but rather used to assign the boolean output.

Question 3. What is meant when you say this is based on ‘patients like you’?

- - Answer: For all patients, we group by cancer type and then look at lines of therapy and prediction status (above or below the threshold for classifying expected survival as low or likely).

Additional questions to address later:

- - How often should the observed population survival data be updated?

# General layout and design specifications

## Layout


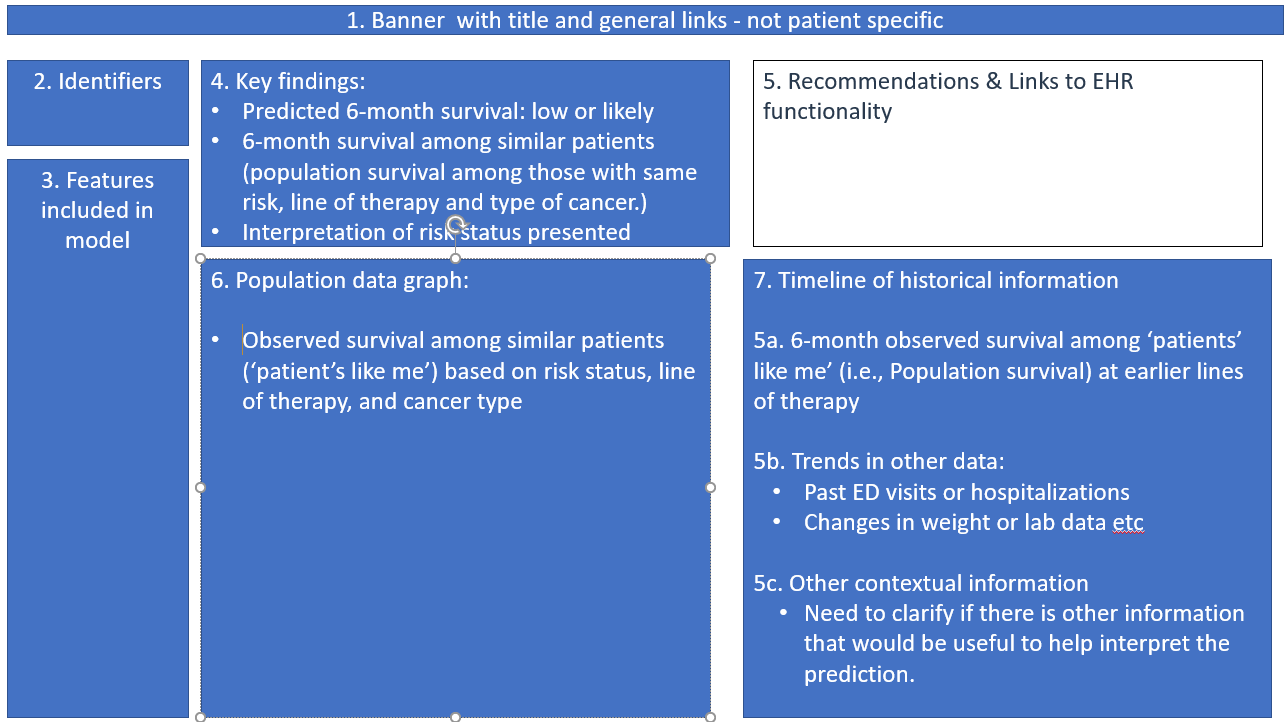


## Specifications to be used throughout the GUI:

1. Use the same colors to indicate low or high risk everywhere, including:
   - block in section 4
   - Bars in section 6 and
   - line and shading in section 7.
2. Only capitalize the first word for all the labels used in the figures.
3. Use white for all text – data elements, values, labels. The only exception is in the Recommendations block.
4. Icons should be meaningful:
   - Section 1. Holding hands in heart and the ‘i’ for more information
   - Section 4. Calendar – today’s prediction
   - Section 5. Speaking block – Recommended actions
   - Section 6. Graph – personalized survival
   - Section 7. People – population survival patterns
5. Use of Color
   - Use color-blind friendly palette of colors to ensure use of colors to communicate information is accessible to all users.
   - In the White Recommended Action section, we can use the standard blue underlined text for links to URLs and the standard ‘infobutton’ icon used in Epic.
   - Where to apply colors:

- Gold: background for header & text for PATIENT, MODEL INPUT, metastases, 3-month % change
- White: all text on dark background, line on the graph
- Green: bad news – low survival block, bar, line
- Purple: good news – high survival block, bar, line
- Grey: error whiskers on bars & background for section titles, and box around section 3, 5, 6
- Black: background in selected areas & text for recommendations
- Red: DO NOT USE


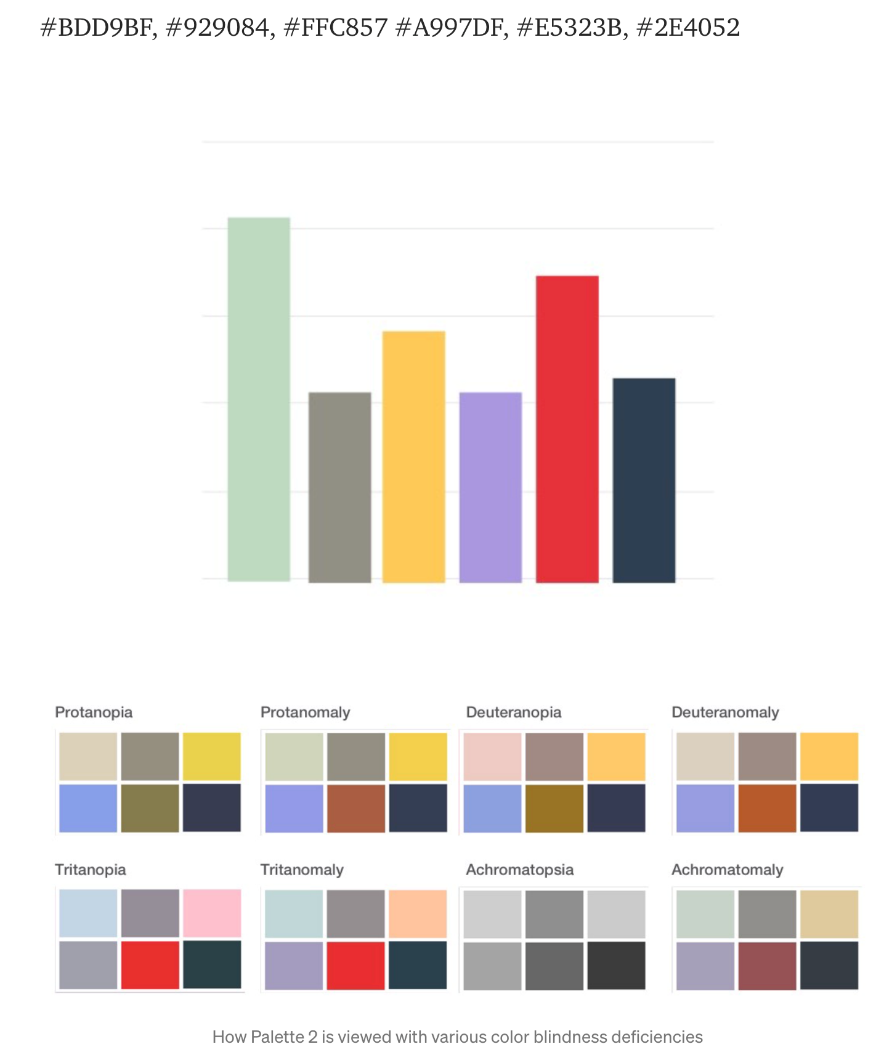


# Description of sections within the GUI

## Section 1. General Banner

Placement: Across the top

### What question is this section answering?

- What is the purpose of this tool?
- Where do I go to get more information?

### Content:

| Label to use in GUI | Sample value |
| --- | --- |
| Icon | Holding hands in heart |
| Text | Next Steps: Support for Shared Decision Making when considering next line of therapy for advanced cancer |
| ‘i’ | Link to additional content about the tool itself |

### Display features:

Sample:

###
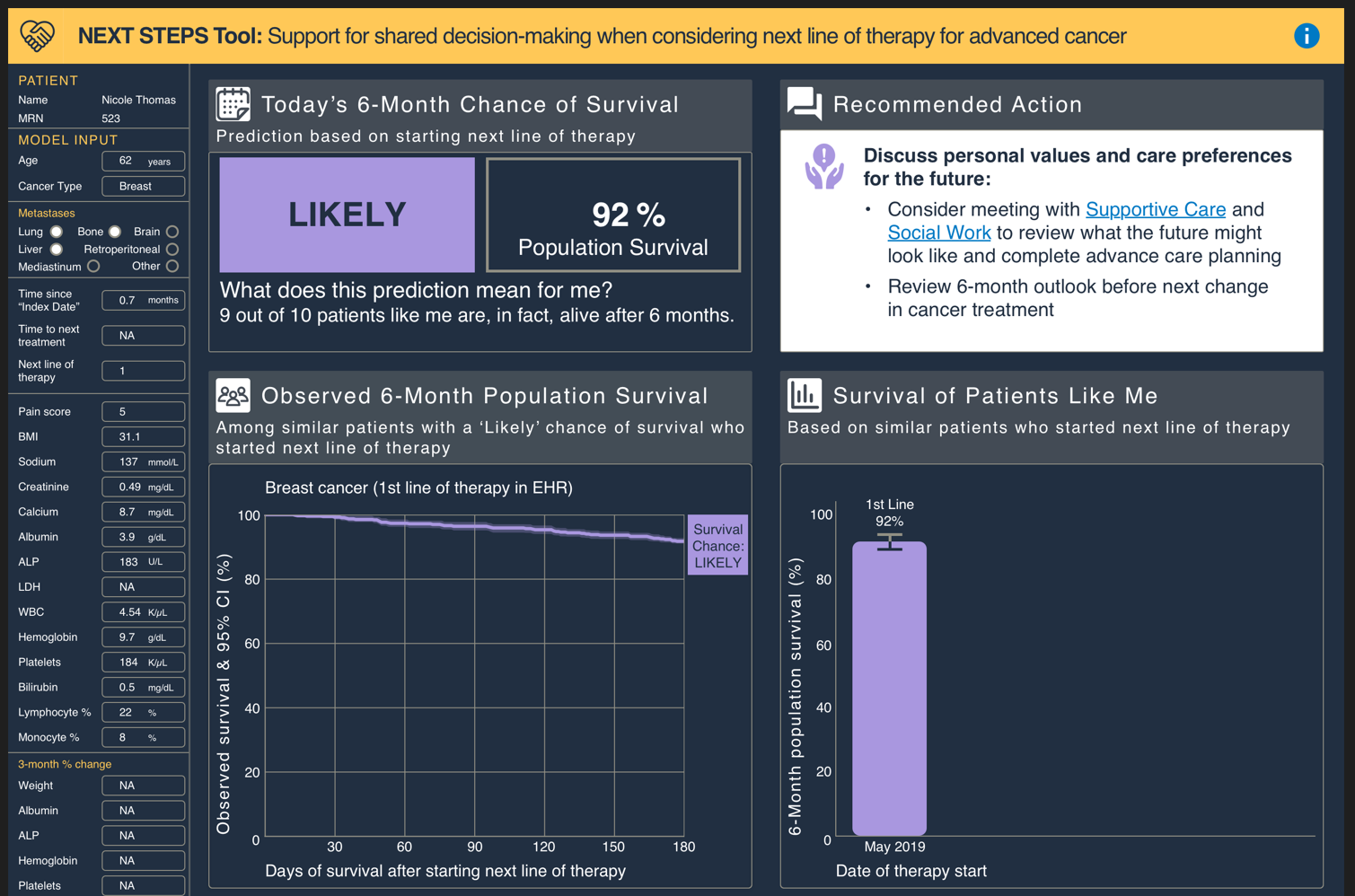


### Questions to consider exploring with users:

- What information is needed on the linked ‘information’ page

## Section 2. Identifiers

Placement: Place top left

NOTE: this section may not be necessary if the interface is part of an EHR-embedded app, such as a SMART-on FHIR app.

### What question is this section answering?

- What patient is the focus of this information?

### Content:

| Label to use in GUI | Sample value | Units |
| --- | --- | --- |
| Name: | Jenny Smith | --- |
| MRN: | 790 | --- |

### Display features:

- Header: PATIENT
- Enclose in a box to indicate content that is displayed, not interactive

## Section 3. Features included in the model (Left panel):

Placement: Place lower left sidebar

### What question is this section answering?

- What data or logic was used to make the prediction?

### Content

| concept | Label to use in GUI | units | comments |
| --- | --- | --- | --- |
| Age | Age | years |  |
| Cancer Type being treated | Cancer Type |  |  |
| Metastasis Categories | Metastases |  | Step 1 – show as a list to view  Future – worry about how to implement a multi-select |
| Time since *index date* | Time since Advanced Cancer Dx | months | Need to calculate months based on days  Need to test with users about what units to use: days, months, years  Use one digit after decimal point |
| Time to next treatment | Time to next treatment |  | Use one digit after decimal point  Use months as unit of measure |
| Next line of therapy | Next line of therapy |  | 1 or 2 etc |
| Pain score | Pain score |  | Ranges from 0-10 |
| BMI | Body mass index | -- | All the BMI measures should just be whole numbers (e.g., 26%, not 26.03%) |
| Sodium | Sodium | mmol/L | Report exactly what was reported – no rolling up |
| Creatinine | Creatinine | mg/dL | Report exactly what was reported – no rolling up |
| Calcium | Calcium | mg/dL | Report exactly what was reported – no rolling up |
| Albumin | Albumin | g/dL | Report exactly what was reported – no rolling up |
| Alkaline Phosphatase | Alkaline Phosphatase | U/L | Report exactly what was reported – no rolling up |
| LDH | Lactate dehydrogenase | U/L | Report exactly what was reported – no rolling up |
| WBC | WBC | K/µL | Report exactly what was reported – no rolling up |
| hemoglobin | hemoglobin | g/dL | Report exactly what was reported – no rolling up |
| Platelets | Platelets | K/µL | Report exactly what was reported – no rolling up |
| Lymphocyte % | Lymphocyte % | % | Report exactly what was reported – no rolling up |
| Lymphocyte # | Lymphocyte # | K/µL | Report exactly what was reported – no rolling up |
| LABEL for sub- section | 3-month % change |  |  |
| Three-month percent weight change | weight | +/- % | decrease or increase |
| Three-month percent albumin change | Albumin | +/- % | decrease or increase |
| Three-month percent weight change | ALP | +/- % | decrease or increase |
| Three-month percent weight change | Hemoglobin | +/- % | decrease or increase |
| Three-month percent weight change | Platelets | +/- % | decrease or increase |

### Display features:

- Header: MODEL INPUT
- Use sub-headers for ‘metastases’ and ‘3-month % change’
- For provider view, display all the features in the order listed above
- For patient view, do not display
- Display reported values provided in the data – e.g. the lab data as reported
- All the data elements and values should be white - do not attempt to flag abnormal values
- For calculated values (time since index date or TTNT), use one digit after the decimal.
- Group the 3-month change features together at the bottom.
  - NOTE: the weight change may be + or – so need to say ‘change’ not ‘loss’
- Step 1 – just display content. The user should not expect to be able to change the values in this list

## Section 4. Key findings

Placement: Place upper middle

### What question is this section answering?

- What is my 6-month chance of survival – likely or low?
- What was the 6-month survival among patient’s like me? (% population survival)
- What does this prediction mean for me? [changing to ask the question as a patient would ask it.]

### Display features:

- Title: Today’s 6-Month Chance of Survival
- Subtitle: Prediction based on starting next line of therapy
- Box 1: placed to the left so seen first
  - Display ‘Likely’ or ‘Low’ based on risk
  - Shade box using low vs likely risk color scheme
- Box 2: placed to the right
  - Line 1: ## % for the relevant risk group
  - Line 2: display ‘Population Survival’ in smaller font
- Text: “what does this prediction mean to me?
  - NOTE: We now phrase it as a patient may ask the question, and show context-specific population survival, based on data for each combination of cancer type and line of therapy, and risk group. A ‘knowledgebase’ would need to be accessed when populating the GUI.
- Add answer based on status:
  - Report actual 6-month survival among patients ‘like me’ (i.e. same risk stratification, cancer type, and line of therapy). For example: “# out of 10 patients like me are, in fact, alive after 6 months.”

### Sample:

NEW: Switched so the statement of Likely or Low (in purple) is on the left side, as that is what we want the patient to consider first.

###
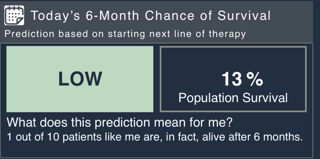

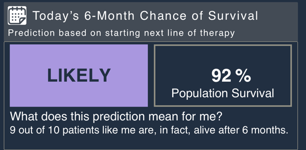


## Section 5. Recommended Action, including logic

Placement: upper right

### What question is this section answering?

- What are the recommended actions?
- Where do I go to implement the recommended actions?

###
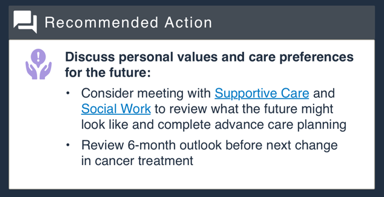
Examples:

###
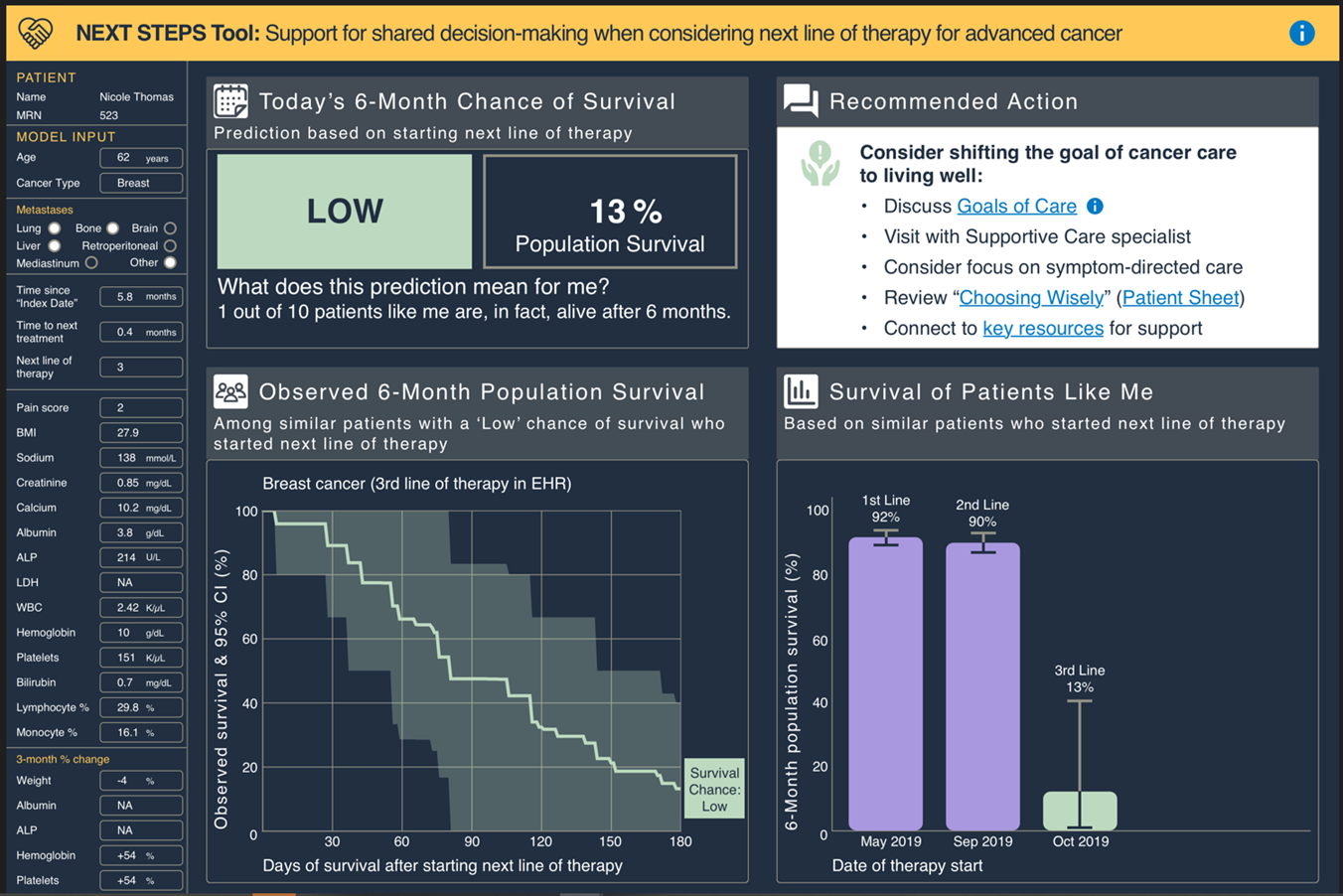


### Display features:

- Display recommendations with a white background so clearly different than the data and increase font to make it stick out
- Step 1 – display the links to simulate that the user should expect to be able to go to the site in the HER or to a weblink.

### Key reference and how we operationalize it:


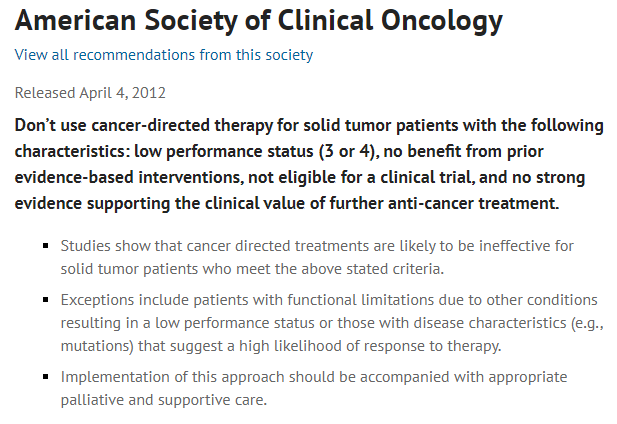


| **Don’t use cancer-directed therapy for solid tumor patients with the following characteristics:** | |
| --- | --- |
| *Choosing wisely Criteria:* | *Could be operationalized by following info:* |
| low performance status (ECOG of 3 or 4) | - Most recent ECOG score - Judgement about quality of life and functional status |
| no benefit from the prior evidence-based interventions | - Time to next treatment (TTNT) which is a surrogate for progression-free survival - Line of therapy Number - Oncologist judgement and knowledge |
| not eligible for a clinical trial | - Availability of a trial - Criteria for Trial eligibility |
| no strong evidence supporting the clinical value of further anti-cancer treatment | - True if predicted chance of survival in next 6 months is LOW (<30%) |

###

### Logic to display recommended action while being consumable for the patient

**IF Likely chance to survive:**

| **6 month expected survival** | **Text and links to display for Recommendation** |
| --- | --- |
| likely | **Discuss personal values and care preferences for the future:**   - Consider meeting with [Supportive Care](https://healthcare.utah.edu/huntsmancancerinstitute/treatment/supportive-oncology-survivorship.php) and [Social Work](https://healthcare.utah.edu/huntsmancancerinstitute/wellness-support/social-workers-support-groups.php) to review what the future might look like and complete advance care planning - Review 6-month outlook before next change in cancer treatment |

**Links:**

- **Supportive Care:** Go to Supportive oncology Website: <https://healthcare.utah.edu/huntsmancancerinstitute/treatment/supportive-oncology-survivorship.php>
- **Social Work:** Go to HCI social work Website: <https://healthcare.utah.edu/huntsmancancerinstitute/wellness-support/social-workers-support-groups.php>


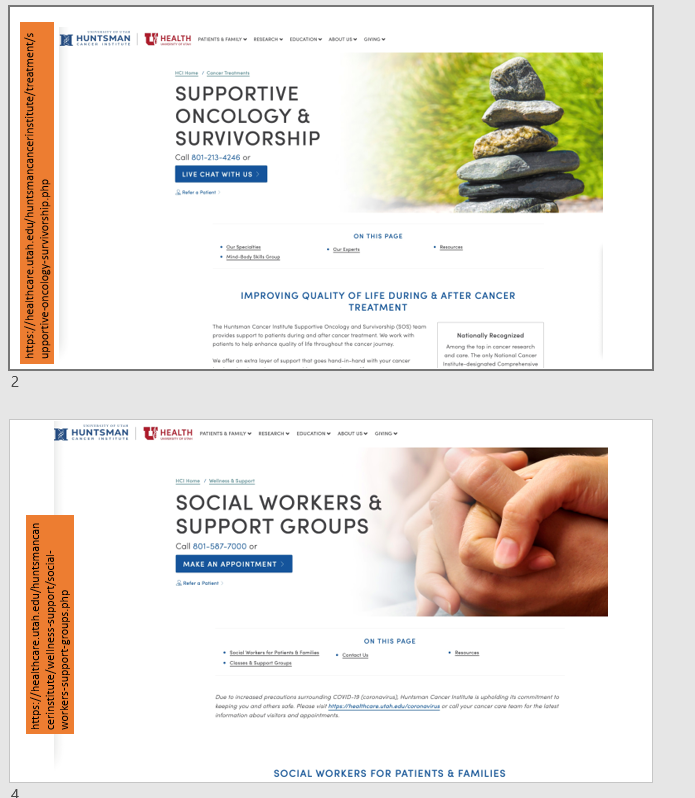


**IF Low chance to survive:**

| **6 month expected survival** | **Text and links to display for Recommendation** |
| --- | --- |
| low | **Consider shifting the goal of cancer care to living well:**   - Discuss [Goals of Care](https://www.ariadnelabs.org/wp-content/uploads/2018/04/Serious-Illness-Conversation-Guide.2017-04-18CC2pg.pdf) - Visit with Supportive Care specialist - Consider focus on symptom-directed care - Review “[Choosing Wisely](https://www.choosingwisely.org/patient-resources/care-at-the-end-of-life-for-advanced-cancer-patients/)” ([Patient Sheet](https://www.choosingwisely.org/wp-content/uploads/2018/02/Care-At-The-End-Of-Life-For-Advanced-Cancer-Patients-ASCO.pdf)) - Connect to [key resources](https://healthcare.utah.edu/huntsmancancerinstitute/treatment/supportive-oncology-survivorship.php) for support |

**Links:**

- **Goals of Care**: Go to the Serious Illness conversation flowsheet in Epic which includes the questions and a picklist and a place to comment for each question. It was implemented a year ago. (can’t include screenshot because can’t share EPIC interface images without permission)
- **Infobutton after Goals of Care**:
  - Go directly to the serious illness conversation sheet: https://www.ariadnelabs.org/wp-content/uploads/2018/04/Serious-Illness-Conversation-Guide.2017-04-18CC2pg.pdf
  - Or Go to the Up-to-Date page re Goals of Care
- **Choosing Wisely**: Go to Choosing Wisely website: https://www.choosingwisely.org/patient-resources/care-at-the-end-of-life-for-advanced-cancer-patients/
- **Patient Sheet**: Go to patient handout: https://www.choosingwisely.org/wp-content/uploads/2018/02/Care-At-The-End-Of-Life-For-Advanced-Cancer-Patients-ASCO.pdf
- **Key Resources:** Go to supportive oncology site: <https://healthcare.utah.edu/huntsmancancerinstitute/treatment/supportive-oncology-survivorship.php>


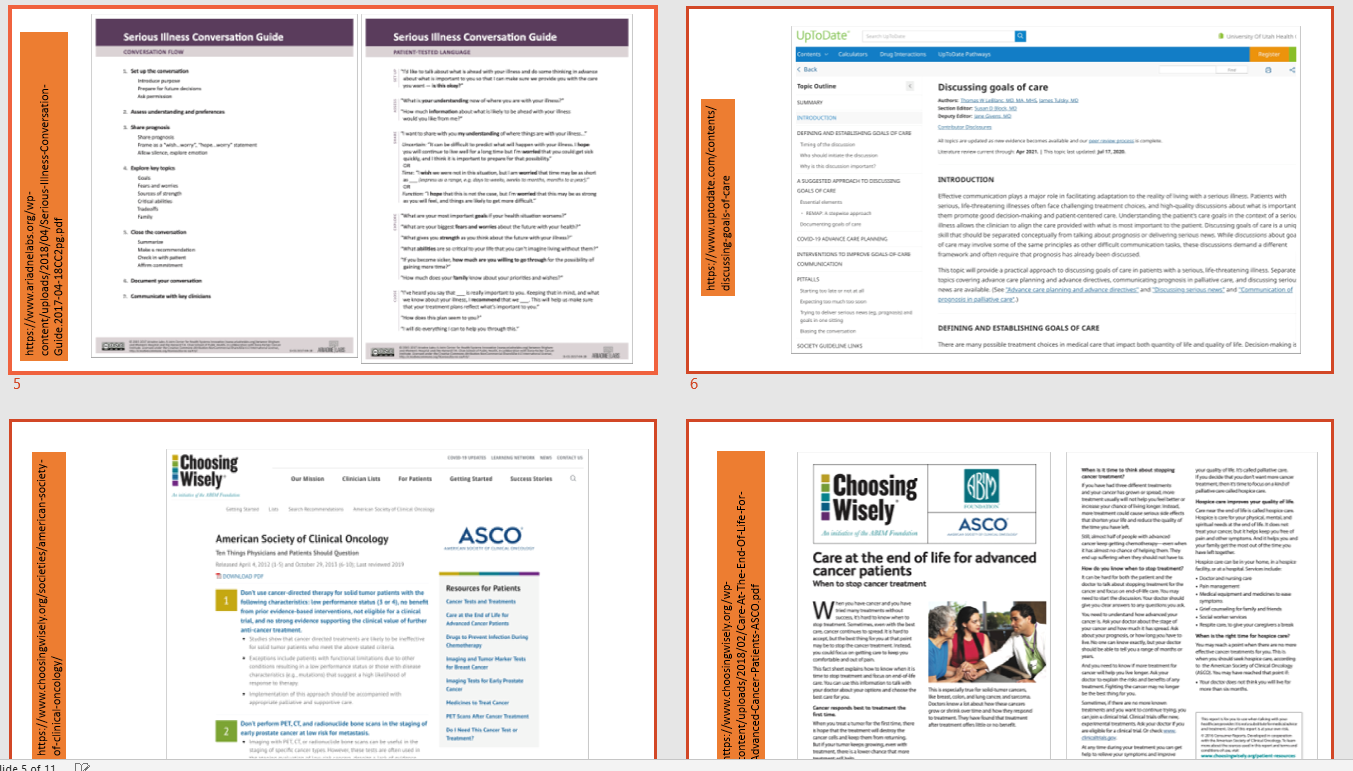


## Section 6. Graphic of Population data illustrating variation

NOTE: Switched these two lower panels based on feedback. Rationale: The history with bar graph makes more sense after the user has seen the line graph.

Placement: lower middle

### What question is this section answering?

### What has been observed for other patients like me, based on risk status, line of therapy, and cancer type.

### How much variance is there in population survival, as shown by confidence intervals?

### Example:


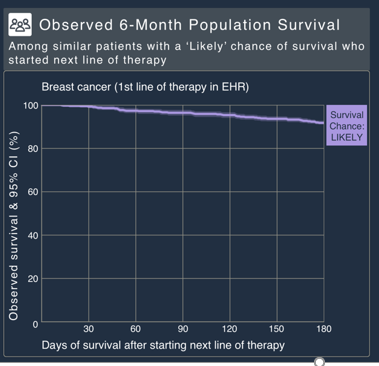


### Display features

- Title
  - first row: Observed 6-Month Population Survival
  - second row (smaller font): Among similar patients with a [‘Low’ or ‘Likely’] chance of survival who started next line of therapy
- X-axis label: Days of survival after starting next line of therapy
- Y-axis label: first line: Observed survival & 95% CI (%)
- Within the graphic:
  - Add text describing ‘patients like me’ (e.g., ‘Lung Cancer (2^nd^ line of therapy in EHR)’)
  - Line label: “Survival Chance: [‘Likely’ or ‘Low’]”
  - X axis label: “Days of survival after starting next line of therapy” with days marked at 0 30 60 80 120 150 180
  - Y axis label: “Observed survival & 95% CI (%)” with (%) marked at 0 20% 40% 60% 80% 100%
  - Use color scheme for line and 95% CI

Future implementation questions:

- - How often should the observed population survival data be updated?
  - How far back should the observed population survival data go to be included?

## Section 7. Timelines of historical information

Placement: lower right

### What question is this section answering?

- Has my predicted survival status and population survival rates changed since last making treatment decisions?
- Where have I been that is leading up to today considering past trends in lab data, weight, and encounters (ED visits, hospitalization)?
  - NOTE: currently, this other info is not included but it could be considered in future

### Example:


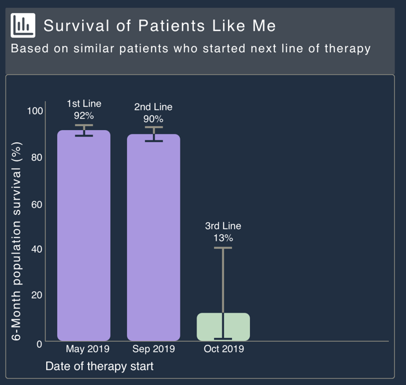


### Display features for trend of past survival status predictions:

- Potentially, this is a tab within this tile, and the user could tab to other contextual info
- Only capitalize the first word for all the labels used in the figure.
- Title:
  - First line: “Survival of Patients Like Me”
  - Second line & smaller font: “Based on similar patients who started next line of therapy”
- X axis:
  - Label axis: Date of therapy start
- bars
  - below the bars: add month/year of the patient’s line of treatment
  - bar is population survival based on risk status of patient (either likely or low)
  - bar color should match the low vs likely risk color scheme
  - above the bar, add ‘1^st^ Line’ and ‘##%’ etc
  - add whiskers to represent the best and worst case scenario for the patient. These values are derived by making predictions using different subsets of the data. After classifying patients into the low and likely groups, and then look at the observed survival for each group. This creates a distribution with a 95% CI that we display as a whisker plot.
  - NOTE: It is okay to use a bar graph (not a time-based histogram) for past lines of therapy, not considering the time because currently MD’s only see it written as text anyway so not on a timeline.
- Y axis:
  - label: “6-Month population survival (%)”
  - (%) marked at 0 20% 40% 60% 80% 100%
- Source of data:
  - To create a display that shows prior population survival statistics, we would need to either:
    - (store a patient’s status (either likely or low) at earlier lines of therapy, OR
    - calculate prior predictions for prior lines of therapy on the fly)
    - AND have access to a knowledgebase that includes the survival rate and confidence interval for different combinations of lines of treatment, prediction status, and cancer types.

# Section 8:
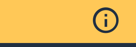
Information page accessed using link in header

**Purpose**

This tool helps you predict the 6-month chance of survival for patients with advanced cancer who are considering the next line of anticancer therapy.

**Description**

The tool derives personalized predictions and observed 6-month survival from Huntsman Cancer Institute (HCI) data, based on patients who underwent anticancer therapy.

**Patient population inclusion criteria:**

Patients with advanced solid tumors* who meet these criteria:

- Date of diagnosis for advanced cancer between June 1, 2014 and June 1, 2020,
- Age 18 years or older when diagnosed with advanced cancer
- No history of hematologic malignancy or bone marrow transplant
- Two or more visits at Huntsman Cancer Institute (HCI) with a medical oncologist from 6 months prior to diagnosis with advanced cancer or later (i.e., being managed at HCI)

*Advanced solid tumors (cancer) are defined as:

- Brain or nervous system cancer (excluding cancers reported as benign, uncertain, in-situ or non-invasive)
- Any solid tumor with a relevant code for metastases documented in the EHR (excluding cancers of the brain and nervous system or those with site histology codes for blood and lymph disorder)

**Treatments used to define line of therapy:**

After diagnosis of advanced cancer, any anticancer therapy (chemotherapy, biologics, targeted therapy, immunotherapy, and next-generation hormonal therapy) entered into a treatment plan in the EHR was used to define line of therapy for this tool. Injectable hormonal therapy and some oral hormonal therapies for prostate and breast cancer were not included.

**Link to publication:** URL here

**Sample Information about Data used to develop and test the predictive model:**

- Last data refresh: Feb 8, 2021
- Eligibility: Index date and patient encounters fall within June 1, 2014 and June 1, 2020.
- Data included for eligible patients: extracted data from 12 months prior to the patient’s index date through November 30, 2020 (allowing for minimum of 6 months follow up for all study patients).

**Version**: 0.1 **Last updated:** 2021-5-25

**Application Development Team**

| Dr. George Chalkidis (Hitachi Ltd.) | Dr. Catherine Staes (U. of Utah) | Dr. Jordan McPherson (HCI) |
| --- | --- | --- |
| Dr. Anna Beck (HCI) | Dr. Teresa Taft (U. of Utah) | Dr. Carolyn Scheese (U. of Utah) |
| Dr. Shuntaro Yui (Hitachi Ltd.) | Wataru Takeuchi (Hitachi Ltd.) | Anastasia Osling (Hitachi Ltd.) |

**Send feedback:** Catherine.Staes@hsc.utah.edu

# References

## Clinical Guideline

Choosing Wisely <https://www.choosingwisely.org/clinician-lists/american-society-clinical-oncology-cancer-directed-therapy-for-solid-tumors>


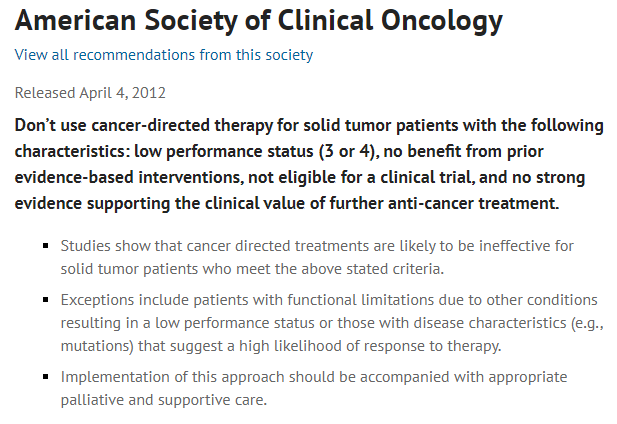


## Quality Measures

American Society of Clinical Oncology (ASCO) Quality Measures related to goal of the tool

| NQF# | Title | Date last updated | Endorsed by NQF |
| --- | --- | --- | --- |
| 0210 | [Proportion of patients who died from cancer receiving chemotherapy in the last 14 days of life](javascript:;) | Oct 25, 2016 | Endorsed |
| 0211 | [Proportion with more than one emergency room visit in the last days of life](javascript:;) | Oct 17, 2016 |  |
| 0212 | [Proportion with more than one hospitalization in the last 30 days of life](javascript:;) | Aug 08, 2012 |  |
| 0213 | [Proportion of patients who died from cancer admitted to the ICU in the last 30 days of life](javascript:;) | Oct 25, 2016 | Endorsed |
| 0214 | [Proportion dying from Cancer in an acute care setting](javascript:;) | Aug 08, 2012 |  |
| 0215 | [Proportion of patients who died from cancer not admitted to hospice](javascript:;) | Oct 25, 2016 | Endorsed |
| 0216 | [Proportion of patients who died from cancer admitted to hospice for less than 3 days](javascript:;) | Oct 25, 2016 | Endorsed |

NQF: National Quality Form

Available from: [NQF search tool](https://www.qualityforum.org/QPS/QPSTool.aspx#qpsPageState=%7B%22TabType%22%3A1,%22TabContentType%22%3A1,%22SearchCriteriaForStandard%22%3A%7B%22TaxonomyIDs%22%3A%5B%5D,%22SelectedTypeAheadFilterOption%22%3Anull,%22Keyword%22%3A%22%22,%22PageSize%22%3A%2225%22,%22OrderType%22%3A3,%22OrderBy%22%3A%22ASC%22,%22PageNo%22%3A1,%22IsExactMatch%22%3Afalse,%22QueryStringType%22%3A%22%22,%22ProjectActivityId%22%3A%220%22,%22FederalProgramYear%22%3A%220%22,%22FederalFiscalYear%22%3A%220%22,%22FilterTypes%22%3A0,%22EndorsementStatus%22%3A%22%22,%22MSAIDs%22%3A%5B%5D%7D,%22SearchCriteriaForForPortfolio%22%3A%7B%22Tags%22%3A%5B%5D,%22FilterTypes%22%3A0,%22PageStartIndex%22%3A1,%22PageEndIndex%22%3A25,%22PageNumber%22%3Anull,%22PageSize%22%3A%2225%22,%22SortBy%22%3A%22Title%22,%22SortOrder%22%3A%22ASC%22,%22SearchTerm%22%3A%22%22%7D,%22ItemsToCompare%22%3A%5B%5D%7D) available here: https://www.qualityforum.org/what_we_do.aspx
