# Supplementary material for: Design of an interface to communicate artificial intelligence-based prognosis for patients with advanced solid tumors: a user-centered approach
Source: J Am Med Inform Assoc. 2023 Oct 17;31(1):174–87. doi: 10.1093/jamia/ocad201 (PMC10746322; doi:10.1093/jamia/ocad201)
Supplement: ocad201_Supplementary_Data [file ocad201_supplementary_data.zip › ocad201_Supplementary_Data/Supplement_eMethods.docx]

Supplement for:

**Design of an Interface to Communicate Artificial Intelligence-Based Prognosis for Patients with Advanced Solid Tumors: A User-Centered Approach**

Contents

[**Interview script** 2](#_Toc135148370)

[**Codebook for qualitative analysis** 5](#_Toc135148371)

## **Interview script**

**1. Introduction**

Thank you for agreeing to participate. Have you had a chance to review the consent letter? <If no “I will pull it up on the screen for you to review”. Do you have any questions?

As a reminder, your participation is voluntary, and you can stop at any time. We will be recording this session for transcription and analysis. We will not be evaluating you or your knowledge in any way. We want to understand how an expert like yourself addresses prognosis and to get your impressions of our prognosis tool. The study has 3 parts. First, we will ask you to recall a patient and describe a prognosis conversation. Second, we will review data for 3‐4 case vignettes using a tool that we are developing to support decision making and ask you to provide feedback. And finally, we will ask for your general perceptions and ratings of the tool.

**2. Critical Incident Interview**

Please take a moment to think back to the last patient with metastatic cancer, for whom you estimated 6‐12 months of remaining survival time. Let me know when you have a patient in mind. We don’t need to know specifics of the case; we will focus on three things only.

*First, how did you communicate the expected survival time to the patient or family?*

*Second, what information do you consider in estimating survival time?*

*Third, what type of graphics or tools did you use when talking with the patient about survival?*

+ 15 min

**3. Case 1 ‐ Cognitive walk through & contextual inquiry**

Imagine that you will be meeting separately with 3 patients today to discuss expected survival and to make decisions regarding initiating another line of cancer controlling treatment. Please read the case vignette for patient <Name>. <wait until participant indicates that they are done reading> Next, we will show you a decision support tool for this case and ask you to think aloud as you look at it. Please say any thoughts that come into your mind. You can talk about what you see, what it means, what you can do with it, what you like or don’t like or anything that is confusing, etc. An example of how I might think aloud if I was looking at an online clothing store is, I might say, “I see a list of items for sale and the prices. It looks like baby clothes. I think if I click on the picture, I will be able to order it. I would like to sort these by price but I can’t see any way to do that.”

<Advance to DISPLAY>

<When the participant asks for more information, show the information page> *<Prompt as needed to help the participant ‘think aloud’>*

*‐‐ What do you see?*

*‐‐ What does it mean?*

*‐‐ What can you do with it?*

*‐‐ Is anything confusing or surprising?*

*‐‐ What do you like or dislike?*

**Sections (1‐6)**

Next, we will focus more closely on each section. Please talk about the section in the red

box as you continue to think aloud. We will skip over any sections that you have already

talked about.<Prompt as needed to help the participant ‘Think Aloud.”>

Complete the Case

The next slides will illustrate what may happen over time as this patient progresses and

comes back to see you to consider next steps. When we come to the display, please speak aloud your thoughts. Talk about anything significant or surprising that you see in the display.

<Stop when prognosis changes (ie. low to good) to explore change in recommended actions>

1. *What are your thoughts about this graphic & the new recommended actions?*
2. *Did you notice that they changed?*
3. *What do you think caused the recommended actions to change?*
4. *Please pretend that I am this patient and use the DISPLAY with me to discuss prognosis and next steps.*

**4. Cases 2 & 3 Contextual Inquiry [if time allows]**

Now we will look at a specific point in time for 2 more cases to illustrate how the display

appears with different patient scenarios. As we go through the case please tell us when

you want to advance to the next screen. 1. When we come to the DISPLAY, please talk about anything significant or surprising that you

see.

+45 min

**5. Follow‐up Questions**

Utility

- In what situations would this tool be useful in your practice?
- Do you have any concerns about using a tool like this?

Clarity

- Is anything confusing?
- Does this tool include the information that affects advanced cancer pt. prognosis, as shown in the left panel? What is missing?
- Is anything extra that should be removed?

Trust

- Do the results of the display appear to be valid?
- What would increase your trust in the tool?
- Would you have any concerns if a patient had received therapy elsewhere that wasn’t reflected in this display?

Impact

- How would seeing this display influence your decision about recommending another line of anti‐cancer therapy?
- Comments

Do you have any other recommendations or comments?

**6. Tool Rating (asked during the final 10 interviews)**

Finally, we will ask you to rate the tool regarding specific qualities on a scale of 1 low to 7 high.

- **How useful is the tool?**
  - - Low Usefulness 1 2 3 4 5 6 7 High Usefulness
- **How much do you trust the information in the tool?**
  - - Low Trust 1 2 3 4 5 6 7 High Trust
- **How much do you agree with the recommended actions?**
  - - Low Agreement 1 2 3 4 5 6 7 High Agreement
- **How much impact would seeing this display have on your confidence in shared decision-making conversations about next steps?**
  - - Low Impact 1 2 3 4 5 6 7 High Impact
- **How likely are you to use this tool when considering a next line of therapy?**
  - - Low Likelihood 1 2 3 4 5 6 7 High Likelihood
- **How likely would you be to recommend this tool to colleagues?**
  - - Low Likelihood 1 2 3 4 5 6 7 High Likelihood
- **How well does the tool support your awareness of the patient’s prognosis?**
  - - Low Support 1 2 3 4 5 6 7 High Support
- **How well does the display align with what you would expect to see?**

Low Alignment 1 2 3 4 5 6 7 High Alignment

**7. Demographics**

Finally, we have 3 demographic questions.

- How many years have you worked in Oncology?
- What is your specialty?
- What gender do you identify with?

## **Codebook for qualitative analysis**

| **Usability Domain** | **Definition** | **Sub-codes** |
| --- | --- | --- |
| Match between the system and the real world | The system should use words, phrases and concepts familiar to the user, rather than system-oriented terms. Follow real-world conventions, making information appear in a natural and logical order | - M01_the system DOES NOT speak the user’s language and visual understanding - M02_information DOES NOT appear in a natural and logical order - M03_process DOES NOT match workflow - M04_available options DO NOT match needs of the user - M05_information is NOT delivered at the right time to the right person - M06_degree of detail is NOT appropriate for the intended user - M08_links are NOT easily identified - M_Positive |
| Trust & Transparency | Trust in the system should be supported by transparency and disclosure of relevant information | - T01_developers or stewards are NOT listed - T02_intended user is NOT clearly identified - T03_goals of use are NOT clearly stated - T04_date of development or last update is NOT clear - T06_contact information is NOT listed with an invitation to give feedback for improvement - T07_source of information is NOT clear - T08_information presented is NOT reliable - T_Positive |
| Aesthetic and minimalist design | Every extra unit of information in a dialogue competes with relevant units of information and diminishes their relative visibility. | - A01_sequences of action are NOT clear - A02_presentation of information is NOT efficient and concise - A03_font sizes are NOT large enough for easy reading - A04_text DOES NOT high contrast from background - A05_first word in menu choices is NOT most important - A07_there are PROBLEMS for colorblind - A11_PROBLEMS on use of color aids comprehension - A09_PROBLEMS on visual display of information, such as charts and graphs aid interpretation - A13_pictures, arrows, labels or other visuals DO NOT give clues to use - A_Positive |
| Recognition rather than recall | Minimize the user's memory load by making objects, actions, and options visible. Users should not have to remember information from one part of the process to another. Instructions for use of the system should be visible or easily retrievable | - R01_instructions are necessary - R02_annotations and footnotes DO NOT provide detail information about decisions or processes - R05_headings DO NOT convey essential information in the first 4 words - R06_all necessary information is NOT visible - R_Positive |
| Consistency and standard | Users should not have to wonder whether different words, situations, or actions mean the same thing. Standards and conventions in product design should be followed. | - C01_DO NOT consistent use of color and shape to communicate context - C02_layout and position DO NOT have spatial consistency - C03_font use is NOT consistent (levels) - C04_DO NOT follow language conventions, terminology, spelling, capitalization - C06_DO NOT present standard position & usage of elements - C07_degree of detail is NOT consistent - C08_shapes DO NOT have consistent meaning and follow conventions - C10_meaning of icons is NOT clear and consistent - C13_labels DO NOT reflect the content of their section - C_Positive |
| Help and documentation | Even though it is better if the system can be used without documentation, it may be necessary to provide help and documentation. Any such information should be easy to search, focused on the user's task, list concrete steps to be carried out, and not be too large | - H01_instructions are NOT visible or easily accessed - H02_DO NOT clearly focus on the user's task - H03_DO NOT List concrete steps to be carried out - H04_it Is NOT concise and easy to follow - H06_use of the system is NOT intuitive - H_Positive |
