# Supplementary material for: Design of an interface to communicate artificial intelligence-based prognosis for patients with advanced solid tumors: a user-centered approach
Source: J Am Med Inform Assoc. 2023 Oct 17;31(1):174–87. doi: 10.1093/jamia/ocad201 (PMC10746322; doi:10.1093/jamia/ocad201)
Supplement: ocad201_Supplementary_Data [file ocad201_supplementary_data.zip › ocad201_Supplementary_Data/Supplement_Heuristic evaluation.docx]

**Instructions for performing the Heuristic Evaluation**

1. Select a team of 2-5 reviewers with varied background and experience including a usability expert and subject matter expert.
2. Train inexperienced reviewers on the goals of each heuristic. Show example violations and provide a practice round on a different tool.
3. Determine the targetted tasks to be completed to evaluate the tool, based on stated purpose of the tool, needs assessment and confirmation with experts
4. Explain the high-level tasks to be performed using the tool, but do not demonstrate or teach how to do them.
5. Independently, explore the tool.
6. Independently, complete the assigned tasks, tracking usability issues as they are encountered, in the row describing the violation, using this form.
7. Describe any issues that do not fit the checklist in the empty rows. Add more rows as needed.
8. Independently, review each line of the checklist and score how well the standard was met.
9. Come together as a team to compare and discuss findings and reach consensus scoring using the Scoring Key.
10. Note action items to address each issue.

**Scoring Key**

0. Standard Met. (No Problem.)

1. Test with users. (Appears to meet standard but verification through user testing is needed.)

2. Minor. (Consider fixing to improve efficiency and/or user experience. Not a functional problem.)

3. Moderate. (Important to fix. May reduce trust, lead to frustration, and/or discourage adoption.)

4. Major. (Imperative to fix before releasing product. May lead to task failure or serious error.)

NA. (Not Applicable.)

**References**

- Kushniruk, A. W., & Patel, V. L. (2004). Cognitive and usability engineering methods for the evaluation of clinical information systems. Journal of Biomedical Informatics, 37(1), 56-76.
- Nielsen J. Fremont: Nielsen Norman Group.; 10 usability heuristics for user interface design. Accessed online on March 5, 2015 at www.nngroup.com/articles/ten-usability-heuristics/. [Google Scholar]
- Nielsen, J. (1994). Heuristic evaluation. In Nielsen, J., & Mack, R. L. (Eds.), Usability Inspection Methods (pp. 25-62). John Wiley & Sons.
- Taft T, Staes C, Slager S, Weir C. Adapting Nielsen’s Design Heuristics to Dual Processing for Clinical Decision Support. AMIA Annu Symp Proc. 2016;2016:1179-1188.
- Zhang, J., Walji, M. F., & Caraballo, K. M. (2018). Heuristic evaluation of clinical decision support systems: A comparison of two usability experts. International Journal of Medical Informatics, 114, 16-22.

**Scoring Checklist**

| **0. Standard Met** | **1. Test with Users** | **2. Minor** | **3. Moderate** | **4. Major** | **N/A Not Applicable** | **Adapted from**  **Nielsen’s Heuristics** | | **Relevant Action Items** |
| --- | --- | --- | --- | --- | --- | --- | --- | --- |
| **A. Aesthetic and minimalist design**  Every extra unit of information in a dialogue competes with relevant units of information and diminishes their relative visibility. | | | | | | | | |
|  |  |  |  |  |  | A1 | Sequences of action are clear |  |
|  |  |  |  |  |  | A2 | Presentation of information is efficient and concise |  |
|  |  |  |  |  |  | A3 | Font sizes are large enough for easy reading |  |
|  |  |  |  |  |  | A4 | Text has high contrast from background |  |
|  |  |  |  |  |  | A5 | First word in menu choices is most important |  |
|  |  |  |  |  |  | A6 | Need for scrolling is obvious if necessary |  |
|  |  |  |  |  |  | A7 | No problems for colorblind |  |
|  |  |  |  |  |  | A9 | Visual display of information, such as charts and graphs aid interpretation |  |
|  |  |  |  |  |  | A10 | Only essential information is collected |  |
|  |  |  |  |  |  | A11 | Use of color aids comprehension |  |
|  |  |  |  |  |  | A12 | Flashing text or icons are avoided |  |
|  |  |  |  |  |  | A13 | Pictures, arrows, labels or other visuals give clues to use. |  |
| **C. Consistency and standards**  Users should not have to wonder whether different words, situations, or actions mean the same thing. Standards and conventions in product design should be followed. | | | | | | | | |
|  |  |  |  |  |  | C1 | Consistent use of color & shape to communicate context |  |
|  |  |  |  |  |  | C2 | Layout and position has spatial consistency |  |
|  |  |  |  |  |  | C3 | Font use is consistent (levels) |  |
|  |  |  |  |  |  | C4 | Language conventions, terminology, spelling, capitalization |  |
|  |  |  |  |  |  | C5 | Keywords, labels are consistent across screens |  |
|  |  |  |  |  |  | C6 | Standard position & usage of elements (e.g., blue underlined text for hyperlink, BPA heading conveys information) |  |
|  |  |  |  |  |  | C7 | Degree of detail is consistent |  |
|  |  |  |  |  |  | C8 | Shapes have consistent meaning and follow conventions |  |
|  |  |  |  |  |  | C9 | Quit, Exit, or Done is located at the bottom right of the screen |  |
|  |  |  |  |  |  | C10 | Meaning of icons is clear and consistent |  |
|  |  |  |  |  |  | C11 | The use of all caps is avoided |  |
|  |  |  |  |  |  | C12 | Menu choices are titles in lower-level menues |  |
|  |  |  |  |  |  | C13 | Labels reflect the content of their section |  |
| **E. Error prevention, recognition,** **diagnosis & recovery**  The systems is a carefully designed to prevent problems from occurring. When error-prone conditions cannot be eliminated. For risky situations, users are presented with a confirmation option before they commit to the action. Error messages should be expressed in plain language (no codes), precisely indicate the problem, and constructively suggest a solution. | | | | | | | | |
|  |  |  |  |  |  | E1 | Interface makes errors impossible |  |
|  |  |  |  |  |  | E3 | The system functions as intended |  |
|  |  |  |  |  |  | E4 | Error messages use clear language and are precise (not system error #8739) |  |
|  |  |  |  |  |  | E5 | Error messages are polite (not “fatal”, “failure”, “illegal”...) |  |
|  |  |  |  |  |  | E6 | Error messages indicate the recovery process |  |
| **F. Flexibility and efficiency of use**  The system can be tailored to accelerate performance according to preferences and experience level. | | | | | | | | |
|  |  |  |  |  |  | F1 | Allows users to tailor frequent actions |  |
|  |  |  |  |  |  | F2 | Shortcuts for experienced users |  |
|  |  |  |  |  |  | F3 | Shortcuts and tailoring are assisted by the system—simple to implement |  |
|  |  |  |  |  |  | F4 | Tedious sequences are avoided |  |
|  |  |  |  |  |  | F5 | Typing requirements are minimal |  |
| **H. Help and documentation**  Even though it is better if the system can be used without documentation, it may be necessary to provide help and documentation. Any such information should be easy to search, focused on the user's task, list concrete steps to be carried out, and not be too large. | | | | | | | | |
|  |  |  |  |  |  | H1 | Instructions are visible or easily accessed |  |
|  |  |  |  |  |  | H2 | Clearly focuses on the user's task |  |
|  |  |  |  |  |  | H3 | Lists concrete steps to be carried out |  |
|  |  |  |  |  |  | H4 | Is concise & easy to follow |  |
|  |  |  |  |  |  | H5 | Clear prompts for data entry |  |
|  |  |  |  |  |  | H6 | Use of the system is intuitive and does not require training to learn to use it |  |
| **M. Match between system and the real world**  The system should use words, phrases and concepts familiar to the user, rather than system-oriented terms. Follow real-world conventions, making information appear in a natural and logical order. | | | | | | | | |
|  |  |  |  |  |  | M1 | The system speaks the user’s language |  |
|  |  |  |  |  |  | M2 | Information appears in a natural and logical order |  |
|  |  |  |  |  |  | M3 | Process matches workflow |  |
|  |  |  |  |  |  | M4 | Available options match needs of the user |  |
|  |  |  |  |  |  | M5 | Information is delivered at the right time to the right person |  |
|  |  |  |  |  |  | M6 | Degree of detail is appropriate for the intended user |  |
|  |  |  |  |  |  | M7 | Language is respectful |  |
|  |  |  |  |  |  | M8 | Links are easily identified |  |
| **R. Recognition rather than recall**  Minimize the user's memory load by making objects, actions, and options visible. Users should not have to remember information from one part of the process to another. Instructions for use of the system should be visible or easily retrievable. | | | | | | | | |
|  |  |  |  |  |  | R1 | Instructions are unnecessary |  |
|  |  |  |  |  |  | R2 | Annotations and footnotes detail information about decisions or processes |  |
|  |  |  |  |  |  | R3 | Decision criterion is clearly stated |  |
|  |  |  |  |  |  | R4 | Links to supporting documents (evidence base, policies...) are active |  |
|  |  |  |  |  |  | R5 | Headings conveys essential information in the first 4 words |  |
|  |  |  |  |  |  | R6 | All necessary information is visible |  |
|  |  |  |  |  |  | R7 | Decisions, and next steps are easy to follow |  |
| **T. Trust & Transparency**  Trust in the system should be supported by transparency and disclosure of relevant information. | | | | | | | | |
|  |  |  |  |  |  | T1 | Developers/stewards are listed |  |
|  |  |  |  |  |  | T2 | Intended user is clearly identified |  |
|  |  |  |  |  |  | T3 | Goals of use are clearly stated. |  |
|  |  |  |  |  |  | T4 | Date of development or last update is clear |  |
|  |  |  |  |  |  | T5 | Clinical rationale is associated with specific best practices evidence |  |
|  |  |  |  |  |  | T6 | Contact information is listed with an invitation to give feedback for improvement |  |
|  |  |  |  |  |  | T7 | Source of information is clear |  |
|  |  |  |  |  |  | T8 | Information presented is reliable |  |
| **U. User control and freedom**  Users are in control. Does not give users the impression that they are controlled by the system. | | | | | | | | |
|  |  |  |  |  |  | U1 | Users can exit quickly. |  |
|  |  |  |  |  |  | U2 | Undo and redo are supported |  |
|  |  |  |  |  |  | U3 | Users are initiators of action, not responders |  |
|  |  |  |  |  |  | U4 | Users can quickly insert themselves at the point they would like in the pathway |  |
|  |  |  |  |  |  | U5 | Alerts can be turned off |  |
|  |  |  |  |  |  | U6 | Options are shown.  What can be done next is clear |  |
|  |  |  |  |  |  | U7 | References have active links to support access |  |
|  |  |  |  |  |  | U8 | Available options match needs of the user. |  |
|  |  |  |  |  |  | U9 | Interruption are justified by level of importance |  |
|  |  |  |  |  |  | U10 | Users can easily move forward and backward in successive screens |  |
|  |  |  |  |  |  | U11 | Users can enter data in any order they desire |  |
|  |  |  |  |  |  | U12 | Users autonomy is respected |  |
| **V. Visibility of system status**  Users should be informed about what is going on within the system, through appropriate feedback and display of information. | | | | | | | | |
|  |  |  |  |  |  | V1 | Options are shown. What can be done next, where the user can go |  |
|  |  |  |  |  |  | V2 | Process flow is clear. Decisions, and next steps are easy to follow |  |
|  |  |  |  |  |  | V3 | Tasks have a clear beginning and end |  |
|  |  |  |  |  |  | V4 | Results of interactions with the system are predictable |  |
|  |  |  |  |  |  | V5 | Feedback clearly communicates changes implemented by every action |  |
|  |  |  |  |  |  | V6 | Labels & headings clearly convey contents for every dataset |  |
|  |  |  |  |  |  | V7 | Visual feedback shows where cursor is |  |
|  |  |  |  |  |  | V8 | Visual indication of what has been selected (i.e., Bold, color) |  |
|  |  |  |  |  |  | V9 | Navigation in the system is intuitive |  |
| **Additional Issues Encountered** | | | | | | | | |
|  |  |  |  |  |  |  |  |  |
|  |  |  |  |  |  |  |  |  |
